# A comparative analysis of the effect of different drying treatments on the volatile organic compounds of polygonati odorati rhizoma

**DOI:** 10.3389/fphar.2026.1965242

**Published:** 2026-10-15

**Authors:** Haili Tang, Shijia Zhang, Ziran Yu, Qiuye Liu, Yajie Zuo

**Affiliations:** 1 The First Hospital of Hunan University of Chinese Medicine, Hunan University of Chinese Medicine, Changsha, China; 2 Academy of Chinese Medical Sciences, Hunan University of Chinese Medicine, Changsha, China

**Keywords:** drying methods, gas chromatography-ion mobility spectrometry (GC-IMS), polygonati odorati rhizoma, principal component analysis (PCA), volatile organic compounds

## Abstract

**Introduction:**

Polygonati Odorati Rhizoma (POR) is a widely recognized functional food, volatile organic compounds (VOCs) as one of their main active metabolites. Drying is a crucial link in the front-end of the product production chain. There have been no comparison investigations on the effects of different drying methods on the VOCs of POR.

**Methods:**

Gas chromatography-ion mobility spectrometry (GC-IMS) was utilized to detect and analyze the VOCs of POR samples treated with vacuum freeze-drying (VFD), vacuum drying (VD), sun drying (SD), hot-air drying (HAD), and microwave drying (MD). Analytical methods including Principal Component Analysis (PCA), Cluster Analysis (CA), and Partial Least-Squares Discriminant Analysis (PLS-DA) were used.

**Results:**

A total of 56 VOCs were detected. Principal component analysis (PCA), cluster analysis (CA), and partial least-squares discriminant analysis (PLS-DA), consistently showed that the VOCs of VFD- and MD-treated samples were the closest to those of SD samples, whereas VD and HAD differed more markedly. Representative drying-specific markers tentatively identified as (E)-2-heptenal and ethyl hexanoate in SD, 2,5-dimethyl thiophene in HAD, (E)-2-butenal in MD, 2-butanol in VD, and 3-methyl-3-buten-1-ol in VFD were observed.

**Conclusions:**

These results indicate that different drying methods significantly affect the VOC profile of POR. VFD and MD are promising for VOC retention.

## 1 Introduction

Polygonati Odorati Rhizoma (POR) is the dried rhizome of *Polygonatum odoratum* (Mill.) Druce (Asparagaceae) (Committee for the National Pharmacopoeia of P.R. China, 2020). It is a popular functional food that contains hypoglycemic, hypolipidemic, anti-fatigue, and anti-aging properties (Gu et al., 2013; Chen et al., 2001; Shu et al., 2009; Wang et al., 2023b). POR is rich in polysaccharides, steroidal saponins, homoisoflavonoids, and lectins, which are generally considered the main bioactive metabolite. Volatile organic compounds (VOCs) are also present and contribute to its characteristic aroma and pharmacological properties. For such medicine–food homologous materials, functional activity is the ultimate development target; Drying is a key processing step that simultaneously affects the retention of active metabolites and the formation of VOCs. Therefore, understanding the effects of drying methods on VOCs and polysaccharides is a crucial prerequisite for the development of POR products. According to earlier research, different hot-air drying (HAD) temperatures significantly influence the concentration of polysaccharide, with the ordering from highest to lowest content being observed at 50 °C, 60 °C, 70 °C, and 80 °C, respectively (Lin et al., 2011). VOCs are also important active metabolites in POR, primarily by providing a unique aroma and flavor and exerting pharmacological effects, such as antioxidant, anti-inflammatory, and antifungal activity against Penicillium expansum (Yijun et al., 2025). However, few studies have demonstrated the effects of different drying methods on the VOCs of POR.

Gas chromatography-ion mobility spectrometry (GC-IMS) is a technique used for analyzing VOCs characterized by high sensitivity, selectivity, and rapid analytical capability. GC-IMS can efficiently capture a large amount of characteristic information of low molecular weight VOCs, covering typical substances such as ketones, phenols, esters, and aromatic aldehydes, effectively compensating for the sensitivity limitations of traditional gas chromatography-mass spectrometry (GC-MS) in the detection of such metabolites. Compared with the detection shortcomings of GC-MS for some polar low molecular weight metabolites, GC-IMS not only significantly improves the efficiency of fingerprint recognition, but also exhibits excellent detection sensitivity for polar low molecular weight metabolites that are difficult to accurately capture with conventional detection techniques (Du et al., 2026). In the field of food classification and identification, GC-IMS can be used in conjunction with chemometric analysis to rapidly and accurately analyze VOCs in food, create unique flavor fingerprint spectra, and categorize and identify various food types and origins (Dai et al., 2024; Bordiga et al., 2025; Tucci et al., 2025).

Part I states that POR is usually harvested in the fall and processed by removing the fibrous roots, washing, sun drying (SD) until soft, repeated rubbing, and open-air drying until the stiff core is removed, followed by additional SD (Pharmacopoeia of P.R. China, Part I). However, the method of open-air drying is time-consuming and laborious, and there is a risk of mold growth on wet days, severely impacting the medicinal and edible value of POR. Traditional Chinese medicine is currently processed using a variety of practical and affordable techniques, including vacuum freeze-drying (VFD), microwave drying (MD), vacuum drying (VD), HAD, and others (Peng et al., 2024; Yin et al., 2024; Chumroenphat et al., 2025). It is well known that different drying methods inevitably result in flavor differences in the final product, the specific changes in VOCs, characteristic flavor markers, and quantitative differences among drying methods in POR remain poorly understood. In particular, no comparative study has systematically evaluated how SD, HAD, VFD, MD, and VD affect the VOCs of POR.

To fill this gap in the literature, we utilized GC-IMS technology to explore the effects of different drying methods, such as SD, HAD, VFD, MD, and VD, on the VOCs in POR. The specific objectives were to: (1) characterize the VOCs of POR under different drying methods; (2) identify characteristic VOCs that discriminate among drying methods; and (3) provide a reference for the selection of drying methods in subsequent POR product development.

## 2 Materials and methods

### 2.1 Materials

The fresh POR samples (Growth year: 2 years) used in this study were cultivated and collected from Liuyang, Hunan, China, on 20 October 2024. The samples were transported to the laboratory and processed on the same day. The rhizomes were washed, sliced, and randomly divided into five groups for the different drying treatments. The plant material was identified by Prof. Dan Huang at Hunan University of Chinese Medicine, and a voucher specimen (HNUCM2025-YZ01) was deposited in the herbarium of the Hunan University of Chinese Medicine.

### 2.2 Drying procedures

For POR drying, the method described by (Yin et al., 2024) was used with slight modifications. All fresh POR samples were dried until a constant weight was reached. For each drying method, three independent drying runs were performed, each using 500 g of fresh POR rhizomes, and all batches were obtained from the same source lot collected at the same location and time. VFD was performed using a freeze dryer (CHRIST, Hagen, Germany); samples were pre-frozen at −85 °C, and drying was carried out at a shelf temperature of −40 °C and a chamber pressure of 0.01 mbar for 10 h, yielding 248 g of dried sample, with a product temperature of approximately −30 °C during drying, drying loss rate is 50.4%. VD was performed using a vacuum oven (Boxun Medical Biological Instrument Co., Ltd., Shanghai, China) at 60 °C and a chamber pressure of −0.09 MPa for 10 h, yielding 246 g of dried sample, drying loss rate is 50.8%. HAD was performed using an electric convection oven (Zhicheng Analytical Instrument Manufacturing Co., Ltd., Shanghai, China) at 60 °C with an air velocity of 0.4 m/s for 10 h, yielding 251 g of dried sample, drying loss rate is 49.8%. MD was performed using a microwave oven (WB-5, Famouk Co., Ltd., Fuzhou, China) at 560 W in three cycles of 1.5 h each, with a 30-min cooling interval between cycles and a duty cycle of 30 s on/30 s off; the total cumulative irradiation time was 4.5 h on a 500 g load, yielding 247 g of dried sample, and the product temperature at the end of each cycle was approximately 65 °C, drying loss rate is 50.6%. SD was performed under natural sunlight for 8 h per day; after each day of sun drying, the samples were rubbed repeatedly and then returned to open-air drying until the stiff core was removed, followed by further sun drying, according to the Pharmacopoeia of the People’s Republic of China (Part I); this procedure was continued for 4 consecutive days, yielding 252 g of dried sample, drying loss rate is 49.6%, during which the average solar irradiance was approximately 500 W/m^2^, the ambient temperature ranged from 14 °C to 24 °C, and the relative humidity was approximately 70%, with the samples collected and kept indoors at room temperature at night to prevent moisture reabsorption. After drying, all samples were ground into powder and passed through a No. 2 sieve (24 mesh, 850 µm aperture) as specified in the Chinese Pharmacopoeia before analysis. The final moisture contents of the dried samples were 8% (VFD), 9% (VD), 10% (HAD), 9% (MD), and 12% (SD). Water activity was not measured.

### 2.3 Analysis by GC-IMS

A FlavorSpec® gas-phase ion mobility spectrometer, manufactured by G.A.S. in Dortmund, Germany, was utilized to directly assess the VOCs in the dried powders. The GC-IMS analysis was conducted according to the procedure described by (Yin et al., 2024), with slight modifications. The headspace incubation conditions were not specifically validated for the starch- and sugar-rich matrix of POR, and no blank or matrix-free control was included.

Each 0.5 g powder sample was placed into a 20 mL headspace vial and incubated for 15 min at 80 °C; following incubation, 100 µL of the headspace gas was introduced *via* non-shunt injection while rotating the vials at 500 r/min for 20 min (injection needle temperature: 105 °C). The VOCs were then separated using an MXT-WAX capillary column (15 m × 0.53 mm × 1.0 mm, Restek Inc., Edmond, OK, United States of America) and maintained at 60 °C with high-purity N_2_ serving as the carrier gas. Initially, the flow rate was 2.00 mL/min; within 8 min, it was linearly increased to 10.00 mL/min, then to 100.00 mL/min within 10 min, and finally held for 30 min, resulting in a total chromatography runtime of 50 min (injection temperature: 80 °C). For the ion mobility spectrometry analysis, tritium (^3^H) was employed as the reagent ion, with a 53 mm drift tube set to an electric field strength of 500 V/cm at 45 °C. High-purity N_2_ (99.999%) flowed at 75 mL/min under positive ion mode conditions. One GC-IMS injection was performed for each independent drying batch. In total, three independent drying batches were analysed for each drying method. Qualitative identification of VOCs was performed by comparing the measured retention indices (RI) and drift times (Dt) against the GC retention index database (NIST, 2020) and IMS drift time database within the VOCal software. No authentic standards of the target metabolites were used, and no orthogonal GC-MS analysis was performed. Therefore, all metabolite identities reported in this study should be regarded as tentative annotations. For retention index (RI) calibration, a mixture of six n-alkanones (C4-C9) was analysed under the same GC-IMS conditions, and the RI of each detected peak was calculated from its retention time. Qualitative identification was performed by matching the calculated RI and measured drift time (Dt) against the NIST 2020 and IMS databases in VOCal software, and metabolites were considered tentatively identified when both RI and Dt matched.

### 2.4 Relative odor activity value (ROAV) analysis

(Jia et al., 2025) established the relative odor activity value (ROAV) for each metabolite based on its relative content and sensory threshold. ROAV indicates the relative contribution of each VOC to the total aroma. Metabolites with ROAV ≥1 are considered key aroma metabolites. The metabolite with the highest impact had a ROAV of 100. The ROAV for the rest of the metabolites was calculated using the following formula:

$$ROAV=\frac{C_{i}}{C_{\max}}\times \frac{T_{\max}}{T_{i}}\times 100$$

C_i_ and T_i_ represent the relative mass fraction (%) and sensory threshold of each metabolite, respectively. C_max_ and T_max_ denote the relative mass fraction (%) and sensory threshold of the most impactful metabolite, respectively.

### 2.5 Statistical analysis

In VOCal data processing software (G.A.S., Dortmund, Germany, version 2.0.0), plugins such as Reporter, Gallery Plot, and Dynamic principal component analysis (PCA) were employed to analyze three-dimensional spectra, two-dimensional spectra, and fingerprints and perform PCA on VOCs. Before PCA, cluster analysis (CA), and partial least-squares discriminant analysis (PLS-DA), the peak height data were normalised using the default settings of the VOCal software. No additional transformation or scaling was applied. PCA was applied to all POR samples processed using five drying methods (VFD, VD, SD, HAD, MD). Each drying group consisted of three independent drying batches. One GC-IMS injection was performed per batch, and moisture content was measured in triplicate per batch. The RSD of peak areas across the three independent drying batches was calculated for each detected metabolite to reflect between-batch variation. All valid samples were tested by GC-IMS without removing any outliers. A total of 77 peaks were detected by GC-IMS, corresponding to 56 unique chemical structures after collapsing monomer–dimer pairs. For the chemometric analysis (PCA, CA, and PLS-DA), all 77 peaks were used as variables, since monomer and dimer forms represent distinct analytical signals. Additionally, CA and PLS-DA were carried out using TBtools and SIMCA (Version 14.1, Umetrics, Sweden), respectively. CA was performed, where Euclidean distance was applied to calculate the characteristic distance between samples, and the complete linkage method was adopted as the inter-cluster merging criterion. The clustering hierarchical results were displayed in the form of a cladogram with branch labels retained, which can intuitively distinguish the influence differences of different drying methods on the VOCs of POR. The significance of the differences between the means was determined using the Kruskal–Wallis test in R (version 4.5.2), followed by Benjamini–Hochberg FDR correction, effect size (ε^2^) calculation, and bootstrap 95% confidence interval estimation.

## 3 Results

### 3.1 The effect of different drying methods on the appearance characteristics of POR

As shown in Figure 1, the appearance, shape, and color of the samples underwent visual changes under different drying methods. The samples were coded as follows: YZ-01, sun drying (SD); YZ-02, hot-air drying (HAD); YZ-03, microwave drying (MD); YZ-04, vacuum drying (VD); and YZ-05, vacuum freeze-drying (VFD). Compared with sample YZ-01, it can be clearly seen that samples YZ-02 and YZ-04 exhibit the same obvious wrinkling after processing as sample YZ-01; The shrinkage degree of sample YZ-03 is relatively small, and the shape of sample YZ-05 shows almost no shrinkage. Samples YZ-02, YZ-03, YZ-04, and YZ-05 all had varied degrees of yellowing in color compared to YZ-01. SD may cause the sample to shrink and show obvious wrinkles on its surface due to the uneven dehydration and surface case-hardening during SD. HAD tends to induce brittleness in POR samples, accompanied by noticeable browning and shrinkage, as the forced convection of hot air drives uniform and thorough dehydration across the entire sample matrix. After low-temperature treatment, the POR samples only showed slight browning and no significant changes in appearance; in comparison, the morphology and structure of the POR samples after thermal drying treatment underwent varying degrees of change, with wrinkles appearing on the surface of the samples.

**FIGURE 1:**
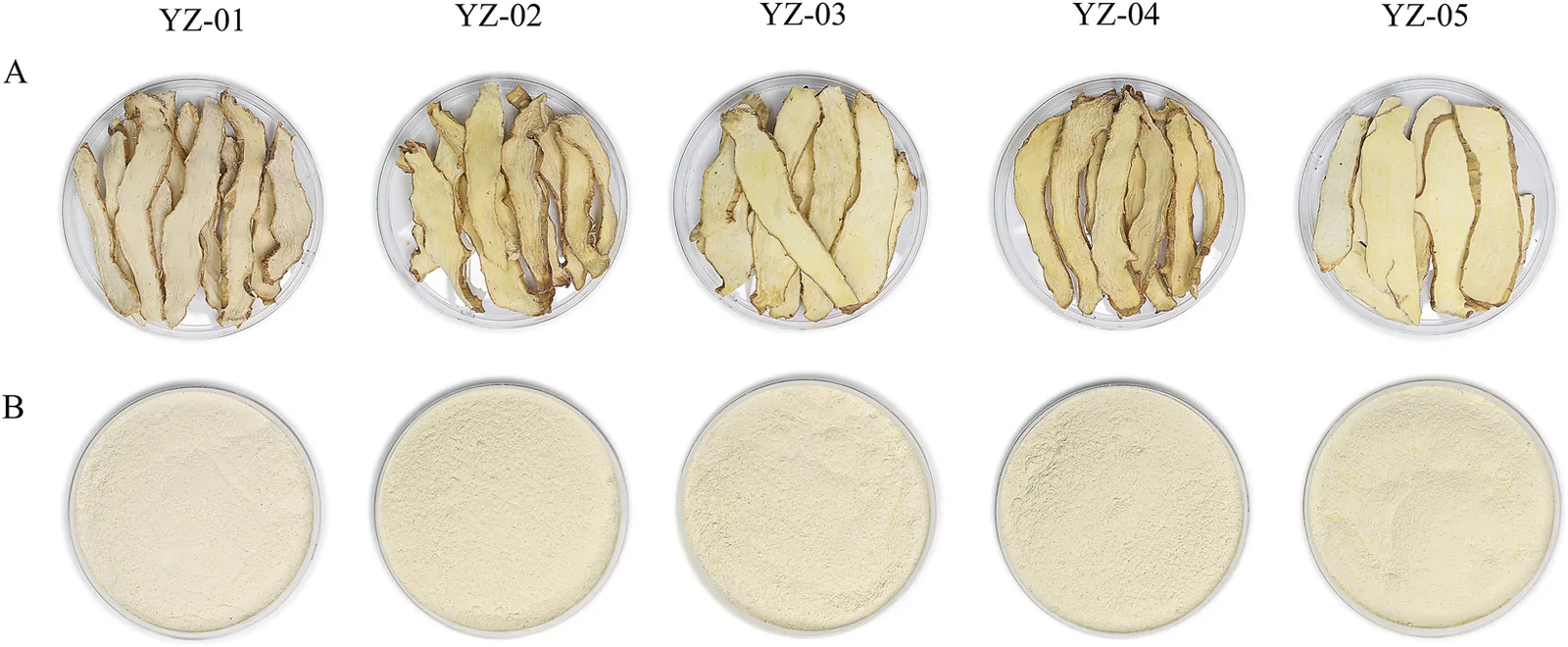
Photos of POR **(A)** and powders **(B)** under different drying methods. YZ-01: SD; YZ-02: HAD; YZ-03: MD; YZ-04: VD; YZ-05: VFD.

### 3.2 Analysis of GC-IMS test results

#### 3.2.1 Comparison of differences in VOCs of POR processed using different drying methods

The three-dimensional spectra produced by the Reporter plugin in GC-IMS analysis software (Figure 2) provide a visual depiction of how VOCs in POR vary across different drying methods. In these spectra, the X-axis represents drift time, the Y-axis denotes gas chromatography retention time, and the Z-axis indicates peak intensity, with each peak corresponding to a distinct VOC. Notably, the height of the red protrusions indicates the relative concentration of these VOCs. As shown in Figure 2, there are pronounced differences in peak position, intensity, and distribution among samples subjected to various drying treatments, highlighting the considerable effect of drying methods on the VOCs of POR.

**FIGURE 2:**
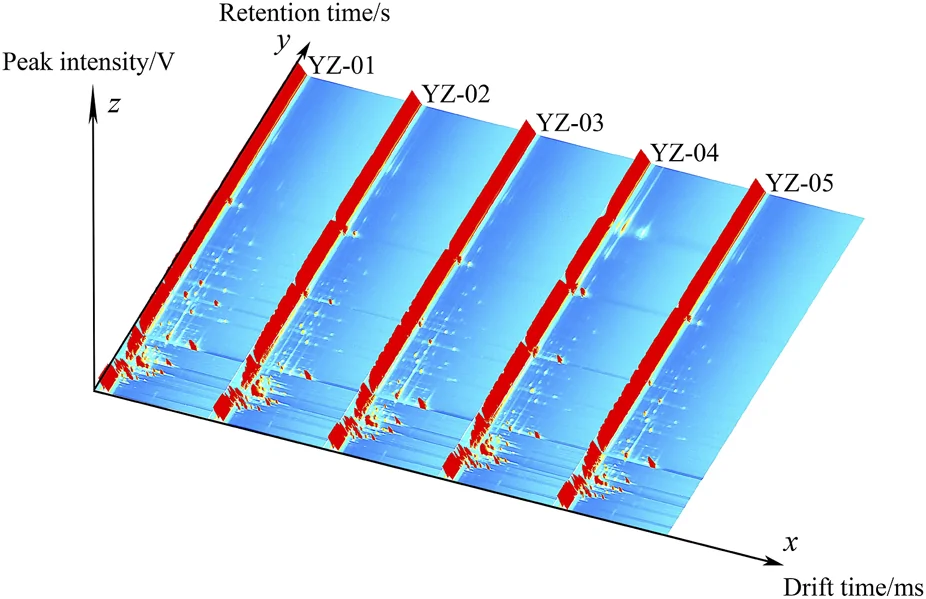
Three-dimensional GC-IMS spectra of VOCs in the POR samples. YZ-01: SD; YZ-02: HAD; YZ-03: MD; YZ-04: VD; YZ-05: VFD.

To facilitate the comparative analysis of intricate 3D data, the spectra’s top view was extracted (refer to Figure 3), enabling a more distinct two-dimensional representation of the differences in VOCs among the samples. This approach clearly illustrated variations in the number of characteristic peaks, shifts in retention times, and changes in intensity.

**FIGURE 3:**
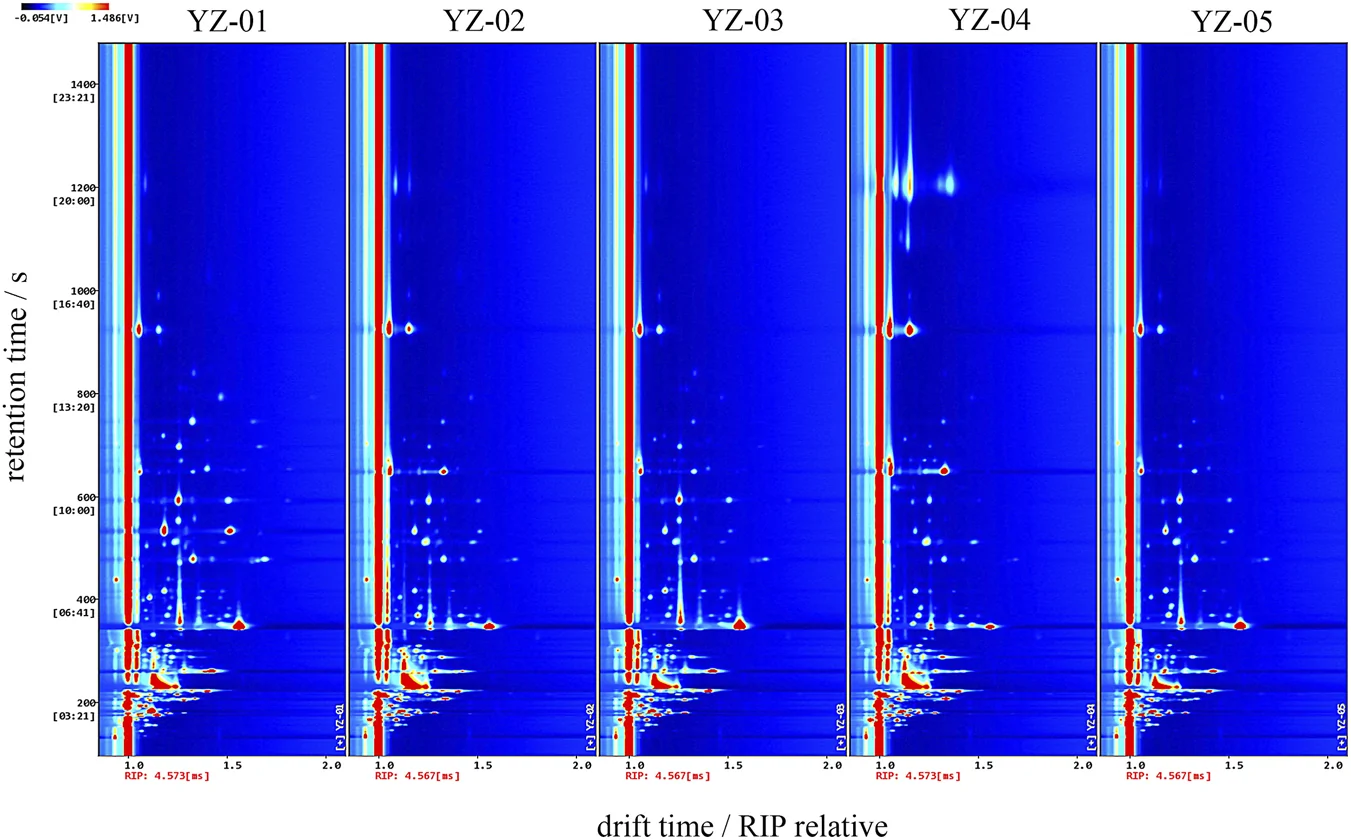
Two-dimensional GC-IMS spectra of VOCs in the POR samples. YZ-01: SD; YZ-02: HAD; YZ-03: MD; YZ-04: VD; YZ-05: VFD.

A comprehensive examination of the GC-IMS data was performed by integrating a two-dimensional top-view plot with an alternative plot. The visualization was rendered against a blue backdrop, with the normalized relative drift time (in arbitrary units) plotted along the horizontal axis to describe ion migration behavior, and the retention time (in seconds) from the gas chromatography separation plotted on the vertical axis. A vertical red line was placed at the 1.0-unit mark on the horizontal axis, indicating the normalized and calibrated Reaction Ion Peak (reference for the characteristic peaks). Scattered points on either side of the RIP represent various VOCs, with color shifts from low-intensity blue to high-intensity red. A two-dimensional map constructed from peak response intensities was used to highlight characteristic features, where a more intense red color indicates a higher detector response for the target metabolite. The two-dimensional spectra clearly reveal that the distribution of VOCs in POR samples differs markedly depending on the drying technique applied.

This study aims to verify whether new technologies such as VFD and MD can achieve highly similar effects in terms of final product quality as traditional SD (limited by natural factors and production efficiency), in order to find an effective alternative to traditional SD methods that are limited by natural factors and production efficiency, and to provide new drying method options for the industrial production of POR. To further compare the characteristics of VOCs resulting from various drying techniques for POR, the spectrum of sample YZ-01 was selected as the baseline. By subtracting the spectra of other samples from this reference spectrum, a comparative chart of differences was generated, as depicted in Figure 4. When a target sample contains the same number of VOCs as the reference, the resulting subtraction yields a white background; red indicates that the target sample has a higher concentration of a specific substance compared to the reference, whereas blue signifies a lower concentration. Notably, for sample YZ-04, the data clearly show that at the same migration time, the concentration of VOCs is markedly higher than that of the reference sample.

**FIGURE 4:**
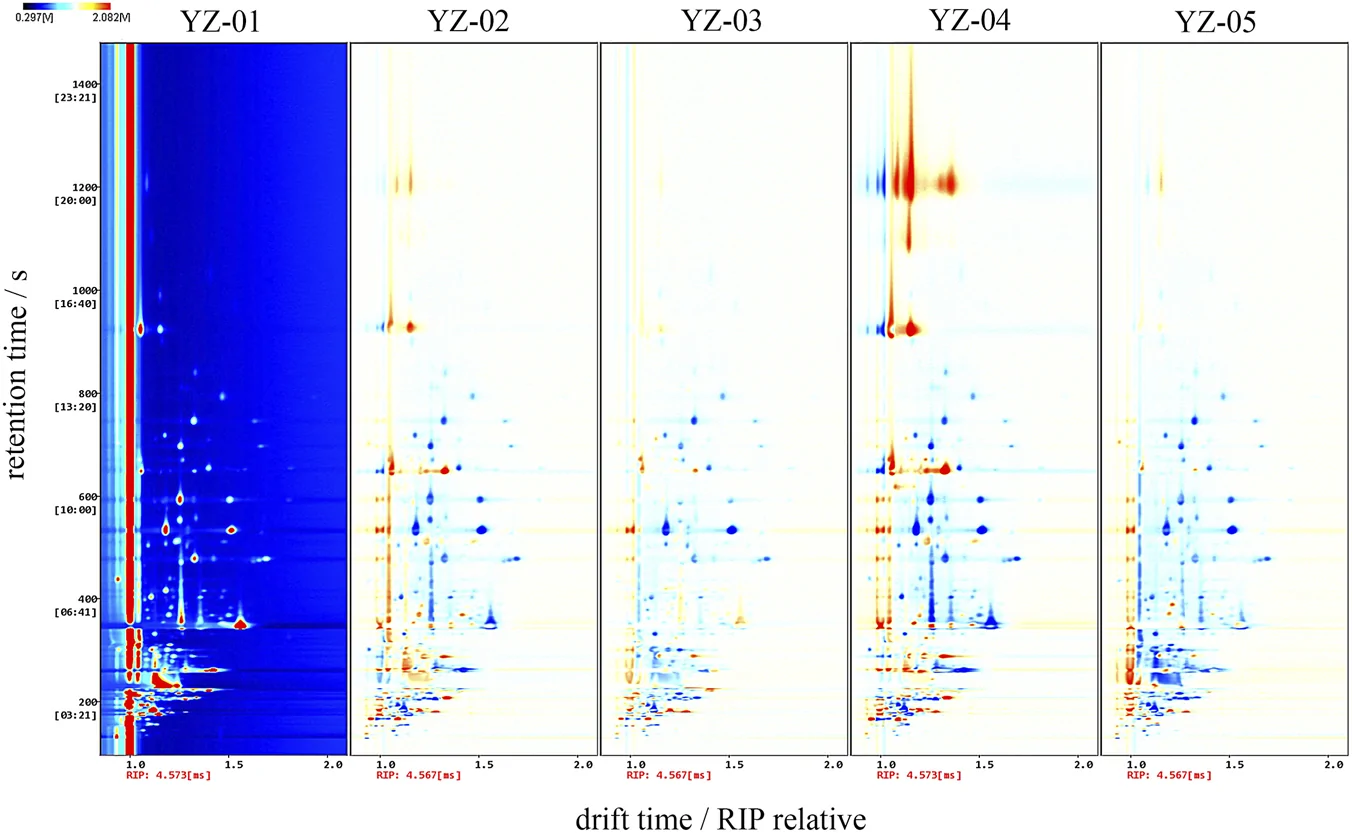
GC-IMS differential spectra of VOCs in the POR samples. YZ-01: SD; YZ-02: HAD; YZ-03: MD; YZ-04: VD; YZ-05: VFD.

#### 3.2.2 Analysis of VOCs in POR using GC-IMS following different drying methods

Using GC-IMS technology to determine the VOCs of samples subjected to different drying methods for POR. A total of 56 VOCs were tentatively annotated in five samples, and the detailed list of VOCs is shown in Table 1. The peak volumes and RSD are provided in Supplementary Table S1. Univariate statistical results (H, p, q, ε^2^, and 95% CI) for all metabolites are provided in Supplementary Table S2. Among them, aldehydes were the most abundant class in terms of metabolite number (18 metabolites, 32.1% of the total identified VOCs), followed by alcohols (14 metabolites, 25.0%), ketones (9 metabolites, 16.1%), heterocyclic metabolites (7 metabolites, 12.5%), esters (6 metabolites, 10.7%), and carboxylic acids (2 metabolites, 3.6%).

**TABLE 1:** The metabolites of VOCs in the POR Samples.

Count	Metabolite	Cas	Formula	MW	RI	Rt [s]	Dt [a.u.]
1	Benzaldehyde	C100527	C_7_H_6_O	106.1	1,509.1	983.977	1.15669
2	Theaspirane	C36431728	C_13_H_22_O	194.3	1,530.5	1,027.128	1.41387
3	Acetic acid-M	C64197	C_2_H_4_O_2_	60.1	1,477.9	924.458	1.05182
	Acetic acid-D	C64197	C_2_H_4_O_2_	60.1	1,476.3	921.483	1.1517
4	Nonanal	C124196	C_9_H_18_O	142.2	1,399.8	790.557	1.4729
5	1-Hexanol-M	C111273	C_6_H_14_O	102.2	1,367.6	741.163	1.32528
	1-Hexanol-D	C111273	C6H14O	102.2	1,370.4	745.397	1.63497
6	(E)-2-Heptenal-M	C18829555	C_7_H_12_O	112.2	1,334.2	693.181	1.25789
	(E)-2-Heptenal-D	C18829555	C_7_H_12_O	112.2	1,336.7	696.709	1.66706
7	1-Octen-3-ol	C3391864	C_8_H_16_O	128.2	1,462.1	895.694	1.16161
8	Ethyl heptanoate	C106309	C_9_H_18_O_2_	158.2	1,343.7	706.588	1.40872
9	1-Octanal-M	C124130	C_8_H_16_O	128.2	1,302.8	650.931	1.40002
	1-Octanal-D	C124130	C_8_H_16_O	128.2	1,303.4	651.715	1.8168
10	1-Pentanol-M	C71410	C_5_H_12_O	88.1	1,267	590.532	1.25358
	1-Pentanol-D	C71410	C_5_H_12_O	88.1	1,265.9	588.571	1.50703
11	2-Pentyl furan	C3777693	C_9_H_14_O	138.2	1,242.2	548.566	1.25499
12	1-Hydroxy-2-propanone-M	C116096	C_3_H_6_O_2_	74.1	1,316.4	668.972	1.05223
	1-Hydroxy-2-propanone-D	C116096	C3H6O2	74.1	1,317.4	670.274	1.22861
13	3-Hydroxy-2-butanone-D	C513860	C4H8O2	88.1	1,299.5	646.639	1.32963
	3-Hydroxy-2-butanone-M	C513860	C_4_H_8_O_2_	88.1	1,298.8	645.832	1.06209
14	(E)-2-Hexen-1-al-M	C6728263	C_6_H_10_O	98.1	1,230.2	529.348	1.18036
	(E)-2-Hexen-1-al-D	C6728263	C_6_H_10_O	98.1	1,229.4	528.172	1.51548
15	1-Butanol, 3-methyl-M	C123513	C_5_H_12_O	88.1	1,216.1	507.601	1.23978
	1-Butanol, 3-methyl-D	C123513	C_5_H_12_O	88.1	1,217.8	510.208	1.49047
16	Butanol-M	C71363	C_4_H_10_O	74.1	1,152	413.827	1.18029
	Butanol-D	C71363	C_4_H_10_O	74.1	1,151.3	412.823	1.37619
17	Ethyl benzene	C100414	C_8_H_10_	106.2	1,139.2	396.09	1.0769
18	Hexanal-M	C66251	C_6_H_12_O	100.2	1,095.3	340.537	1.26464
	Hexanal-D	C66251	C_6_H_12_O	100.2	1,099.3	345.222	1.56121
19	(Z)-2-Pentenal	C1576869	C_5_H_8_O	84.1	1,111.4	359.947	1.09322
20	1-Penten-3-ol	C616251	C_5_H_10_O	86.1	1,168.2	437.64	0.9394
21	Propanal-M	C123386	C_3_H_6_O	58.1	830.9	175.155	1.05343
	Propanal-D	C123386	C_3_H_6_O	58.1	831.5	175.344	1.14634
22	Acetone	C67641	C_3_H_6_O	58.1	856.2	182.845	1.11284
23	Ethanol	C64175	C_2_H_6_O	46.1	957.4	226.967	1.12224
24	2-Butanone	C78933	C_4_H_8_O	72.1	939.4	215.852	1.24776
25	2-Propanol-D	C67630	C_3_H_8_O	60.1	952.2	223.725	1.21866
	2-Propanol-M	C67630	C_3_H_8_O	60.1	953.1	224.304	1.08801
26	3-Methyl butanal-M	C590863	C_5_H_10_O	86.1	952.8	224.072	1.16617
	3-Methyl butanal-D	C590863	C_5_H_10_O	86.1	951.7	223.425	1.40699
27	Acetic acid ethyl ester	C141786	C_4_H_8_O_2_	88.1	925.6	207.696	1.33731
28	Butanal	C123728	C_4_H_8_O	72.1	920.6	204.802	1.28194
29	n-Pentanal-M	C110623	C_5_H_10_O	86.1	1,004.6	259.693	1.17732
	n-Pentanal-D	C110623	C_5_H_10_O	86.1	1,006.2	260.868	1.42202
30	(E)-2-Butenal	C123739	C_4_H_6_O	70.1	1,065.3	310.466	1.20377
31	1-Propanol-M	C71238	C_3_H_8_O	60.1	1,054.6	300.863	1.11132
	1-Propanol-D	C71238	C_3_H_8_O	60.1	1,054.4	300.711	1.25056
32	2-Methyl-1-propanol	C78831	C_4_H_10_O	74.1	1,104.8	351.848	1.17158
33	2-Heptanone-M	C110430	C_7_H_14_O	114.2	1,190.4	470.331	1.26257
	2-Heptanone-D	C110430	C_7_H_14_O	114.2	1,191	471.12	1.6286
34	1-Octen-3-one	C4312996	C_8_H_14_O	126.2	1,316.9	669.632	1.2742
35	Methyl 2-furoate	C611132	C_6_H_6_O_3_	126.1	1,561.4	1,092.762	1.14526
36	2-Furaldehyde	C98011	C_5_H_4_O_2_	96.1	1,429.2	838.456	1.33229
37	(Z)-2-Penten-1-ol	C1576950	C_5_H_10_O	86.1	1,339.6	700.83	0.93884
38	1-Pyrroline, 2-acetyl	C85213225	C_6_H_9_NO	111.1	1,345.8	709.499	1.13267
39	3-Methyl-3-buten-1-ol	C763326	C_5_H_10_O	86.1	1,263.1	583.686	1.16905
40	Ethyl hexanoate	C123660	C_8_H_16_O_2_	144.2	1,248.8	559.468	1.33733
41	3-Methyl-2-butenal	C107868	C_5_H_8_O	84.1	1,214.3	504.972	1.09244
42	Heptaldehyde-M	C111717	C_7_H_14_O	114.2	1,197.2	479.891	1.32633
	Heptaldehyde-D	C111717	C_7_H_14_O	114.2	1,197.2	479.891	1.69389
43	(E)-2-Pentenal-M	C1576870	C_5_H_8_O	84.1	1,145.6	404.848	1.10908
	(E)-2-Pentenal-D	C1576870	C_5_H_8_O	84.1	1,145.2	404.363	1.35791
44	2-Pentanol	C6032297	C_5_H_12_O	88.1	1,132.5	387.048	1.20968
45	2-Hexanone	C591786	C_6_H_12_O	100.2	1,118.1	368.297	1.50034
46	Butyl acetate	C123864	C_6_H_12_O_2_	116.2	1,081.6	325.8	1.23264
47	1-Penten-3-one	C1629589	C_5_H_8_O	84.1	1,044.7	292.227	1.08008
48	2-Butanol	C78922	C_4_H_10_O	74.1	1,040.8	288.9	1.32065
49	Alpha-pinene	C80568	C_10_H_16_	136.2	1,029.3	279.221	1.21503
50	2-Methyl propanal	C78842	C_4_H_8_O	72.1	850.9	181.21	1.28273
51	(Z)-2-Hexenal	R218138	C_6_H_10_O	98.1	1,216.5	508.212	1.181
52	2,5-Dimethyl thiophene	C638028	C_6_H_8_S	112.2	1,186.9	465.429	1.07328
53	Gamma-butyrolactone-M	C96480	C_4_H_6_O_2_	86.1	1,614.1	1,214.19	1.08026
	Gamma-butyrolactone-D	C96480	C_4_H_6_O_2_	86.1	1,612.1	1,209.374	1.30334
54	Propanoic acid	C79094	C_3_H_6_O_2_	74.1	1,566.6	1,104.044	1.10773
55	2-Methyl-3-ketotetrahydrofuran-M	C3188009	C_5_H_8_O_2_	100.1	1,282.7	618.652	1.07306
	2-Methyl-3-ketotetrahydrofuran-D	C3188009	C_5_H_8_O_2_	100.1	1,281.8	617.102	1.41954
56	2-Methyl-2-hepten-6-one	C110930	C_8_H_14_O	126.2	1,349.2	714.35	1.18087

77 peaks were detected, corresponding to 56 unique metabolites after collapsing monomer–dimer pairs; all 77 peaks were used in the chemometric analysis. All identities are tentative annotations based on RI, and Dt matching (NIST, 2020 and IMS, databases). M and D denote monomer and dimer forms. MW: molecular weight; RI: retention index; Rt: Retention time (s); Dt (a.u.): relative migration time.

#### 3.2.3 GC-IMS fingerprint analysis of VOCs in POR treated with different drying methods

The signal peaks and bright spots in the GC-IMS fingerprint spectra are intuitive representations of VOCs. Their locations denote the chemical characteristics, while the varying colors indicate the concentration levels of the metabolites. Specifically, the two-dimensional coordinates of each bright spot align with the unique chemical fingerprint of a VOC, enabling qualitative analysis through comparison with a standard database. Additionally, the gradient of colors reflects the signal intensity, where a deeper red hue suggests a higher peak response, indicative of a greater concentration of metabolites (Parastar et al., 2025; Nicolier et al., 2024).

From Figure 5, the differences in VOCs between several groups of samples are evident. The red boxes indicate the characteristic metabolites of each group of samples, while the black boxes indicate the common metabolites of different samples. For example, the content of alpha-Pinene, acetone, Benzaldehyde, (Z)-2-pentenal, ethyl benzene, 1-Pentanol, 2-pentyl furan, 2-Heptanone, Butanal, 3-Methyl-2-butenal, 2-furaldehyde, (E)-2-Pentenal, Ethyl heptanoate, theaspirane, 1-Octen-3-ol, 1-octanal, Ethyl hexanoate, (E)-2-hexen-1-al, Heptaldehyde, (E)-2-Heptenal, nonanal, 2-methyl-2-hepten-6-one, 1-hexanol, (Z)-2-Hexenal, and other metabolites were found to be more abundant in the YZ-01 sample. The content of 2,5-Dimethyl thiophene was found to be more abundant in the YZ-02 sample. The contents of 3-Methyl butanal, n-Pentanal, Propanal, hexanal, 2-hexanone, 1-Penten-3-one, Butanol, 2-Methyl propanal, and (E)-2-Butenal was found to be more abundant in the YZ-03 sample. The contents of 2-Butanone, 1-Octen-3-one, 1-Pyrroline, 2-acetyl, 1-Hydroxy-2-propanone, acetic acid, 3-Hydroxy-2-butanone, 1-Butanol, 3-methyl, 2-butanol, butyl acetate, 2-Methyl-1-propanol, acetic acid ethyl ester, Propanoic acid, 2-methyl-3-ketotetrahydrofuran, gamma-Butyrolactone, and methyl 2-furoate were found to be more abundant in the YZ-04 sample. The contents of 3-Methyl-3-buten-1-ol, (Z) -2-Penten-1-ol, and 2-Pentanol were found to be more abundant in the YZ-05 sample.

**FIGURE 5:**
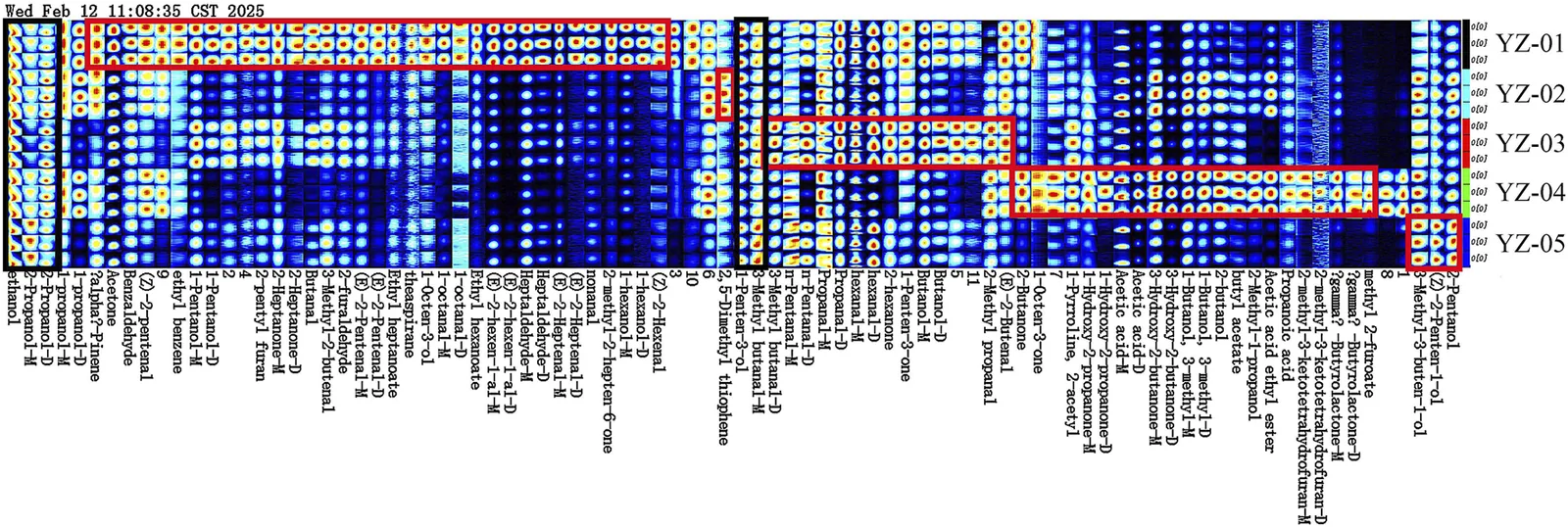
Gallery plot of VOCs selected via GC-IMS. YZ-01:SD; YZ-02: HAD; YZ-03: MD; YZ-04: VD; YZ-05: VFD.

### 3.3 Chemometric analysis

When processing GC-IMS data, we employed three chemometric methods: PCA, CA, and PLS-DA. PCA can be used to transform numerous related original variables into a few orthogonal principal metabolite variables, achieving data simplification. These principal metabolites can be used to reveal similarities and differences between samples (Mareczek et al., 2024). CA and PLS-DA accurately classify samples based on the extracted features (Peng et al., 2024; Yin et al., 2024). This series of methods has been widely applied in many fields, such as traditional Chinese medicine metabolite analysis, agricultural product quality assessment, and food type identification, significantly improving the accuracy and efficiency of data analysis in these fields. Since each drying method corresponded to three independent drying batches, the multivariate patterns should be interpreted as treatment-associated differences rather than as effects of drying treatment alone.

#### 3.3.1 Principal component analysis

The principal metabolites of VOCs in five groups of POR samples were analyzed using VOCal data processing software, and the two-dimensional score plot is shown in Figure 6a. The cumulative contribution rate of the two principals metabolites reach 82%, with PC1 contributing 56% and PC2 contributing 26%. As shown in Figure 6b, the cumulative contribution rate of the three principals metabolites reach 92.3%, with PC1 contributing 55.6%, PC2 contributing 26.1%, and PC3 contributing 10.6%. The 2D PCA plot reports PC1 56% and PC2 26%, while the 3D plot reports PC1 55.6% and PC2 26.1%. This difference is due to rounding; both panels are derived from the same PCA model. The cumulative variance explained by PC1 and PC2 is 82% in both cases. It is evident from the figure that the distance between YZ-01 and YZ-04 is relatively the largest, indicating that there is a significant difference in the content of VOCs in the SD and VD. The distance between the VFD and MD samples is relatively close, indicating that these two drying methods have similar effects on the changes in VOCs in POR.

**FIGURE 6:**
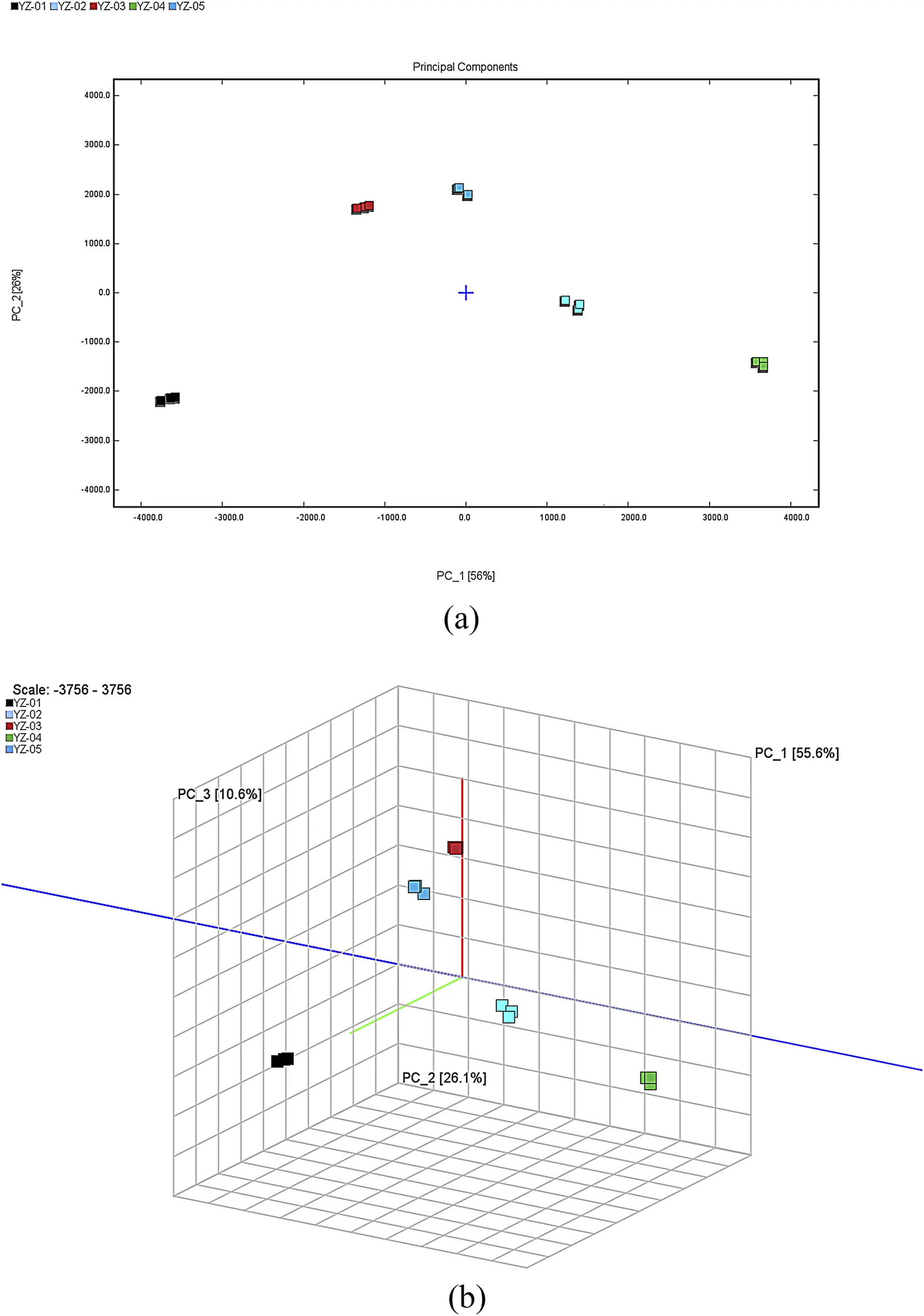
Plot of the PCA scores of VOCs in five species of POR. **(a)** PCA score plot; **(b)** three-dimensional scatter plot. YZ-01: SD; YZ-02: HAD; YZ-03: MD; YZ-04: VD; YZ-05: VFD.

**FIGURE 7:**
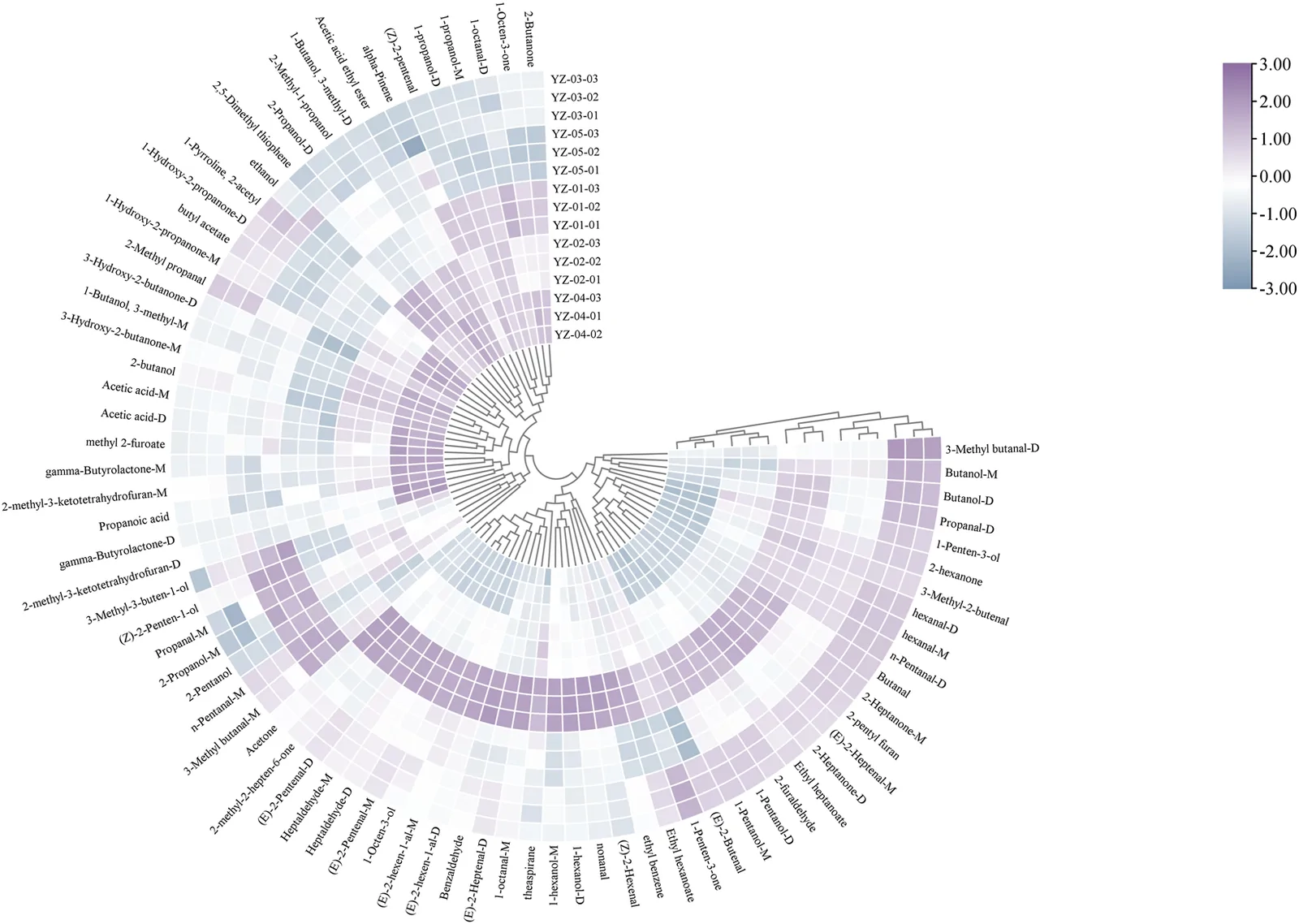
Cluster heat map of VOCs in five species of POR. YZ-01: SD; YZ-02: HAD; YZ-03: MD; YZ-04: VD; YZ-05: VFD.

#### 3.3.2 Cluster analysis

CA is an unsupervised data analysis method that automatically divides a dataset into several groups with similar features by mining the inherent similarities between data, making the data within each group highly similar and the differences between groups significant, to reveal the potential structure and correlations of the data (Scardino et al., 2025; Tsolakou et al., 2025). A heatmap is a visualization tool that represents data distributions or variable associations through color gradients, enabling complex differences to be discerned at a glance. In this study, the peak data of 77 VOCs identified across five groups of POR samples were imported into TBtools software for CA to explore the effect of different drying methods on the composition of VOCs in POR. As shown in Figure 7, the analysis results showed that the VOCs of samples YZ-03 and YZ-05 were the most similar, and these two to be grouped into the same sub-cluster during CA, indicating that the MD and VFD yield highly consistent VOCs compositions. Furthermore, the sub-cluster was subsequently clustered with sample YZ-01, suggesting that the VOC profile of sun-dried POR shares certain similarities with those of MD and VFD, although notable differences remain. Lastly, all samples were clustered together with sample YZ-04 and sample YZ-02 into a large cluster during CA. This clustering pattern clearly indicates that the VOC compositions of POR samples subjected to VD and HAD differ markedly from those samples treated with MD, VFD, and SD, indicating that different drying methods have a significant effect on the formation of VOCs in POR. These findings provide a key chemical basis for a deeper understanding of the influence of drying methods on the quality formation mechanism of POR. In MD, the contents of 1-Penten-3-ol, 2-hexanone, n-Pentanal-D, hexanal-M, hexanal-D, Butanal,1-Penten-3-one, Butanol-D, Propanal-D, Butanol-M, 3-Methyl butanal-D, and 2-Methyl propanal are higher than the other four groups. In SD, the contents of Heptaldehyde-M, (E)-2-Pentenal-M, Heptaldehyde-D, 1-Octen-3-ol, (E)-2-hexen-1-al-D, (E)-2-hexen-1-al-M, (E)-2-Heptenal-M, 2-Heptanone-D, (E)-2-Pentenal-D,theaspirane, E−2-Heptenal-D, Benzaldehyde, 2-methyi-2-hepten-6-one, acetone,1-octanal-M, nonanal, 1-hexanol-M,1-hexanol-D, Ethyl hexanoate,1-Pentanol-M, 1-Pentanol-D, Ethyl heptanoate, 2-furaldehyde, Butanal, and -pentyl furan are higher than in the other groups.

#### 3.3.3 Partial least-squares discriminant analysis

PLS-DA is a multivariate statistical analysis method based on supervised learning, commonly used for classification. This method is suitable for processing high-dimensional data and can preserve the most critical information for classification while reducing the dimensionality of the data. The reliability and predictive ability of the model were evaluated using R^2^ and Q^2^. R^2^ and Q^2^ values greater than 0.5 indicate acceptable model fit, while values closer to one indicate stronger predictive ability (Mu et al., 2024; Theakstone et al., 2021; Sacco Botto et al., 2026; Nath et al., 2025). Using SIMCA software, five sets of sample data were imported: YZ-01, YZ-02, YZ-03, YZ-04, and YZ-05. The results are shown in Figure 8, R^2^X = 0.981, R^2^Y = 0.996, Q^2^ = 0.993. The distance between MD (YZ-03) and FD (YZ-05) is the smallest, indicating that the VOCs in dried POR samples using MD and FD are relatively close. However, there is a significant difference between SD (YZ-01) and VD (YZ-04) compared to the other groups, and in the figure, these two groups are the farthest apart. There is a significant change in the content of VOCs in the dried and vacuum-dried POR samples. The conclusion drawn from the PLS-DA graph is consistent with that drawn from the PCA graph. The PLS-DA model was constructed with two latent variables. The 200-iteration permutation test was performed at the sample-level label permutation hierarchy. Nested cross-validation or held-out-sample classification was not performed in this study.

**FIGURE 8:**
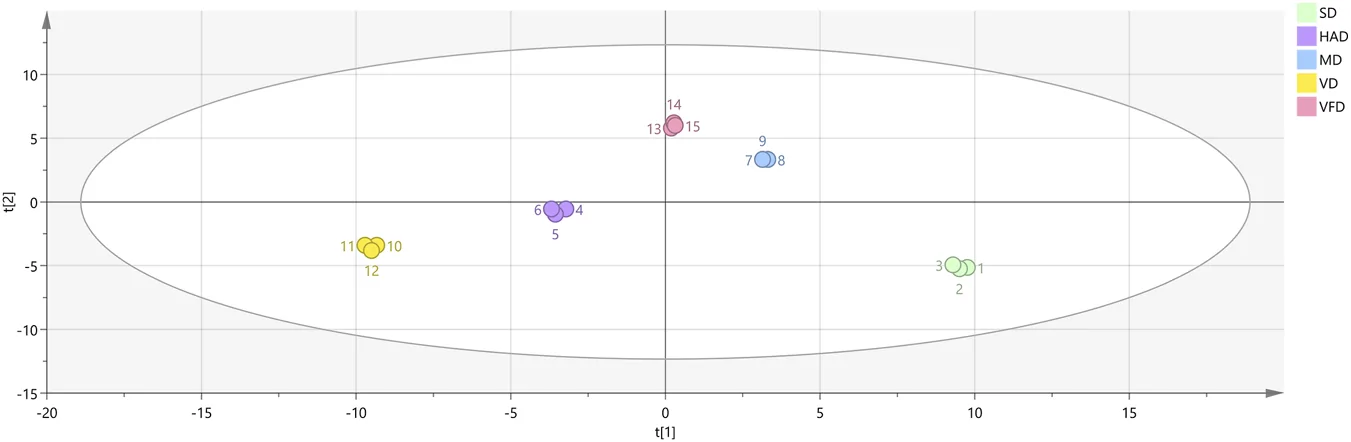
PLS-DA score plot of VOCs in five groups of POR samples.

In chemometrics, VIP (Variable Importance in Projection) measures the contribution of each variable to the PLS-DA model, and variables with VIP ≥1 are generally considered important discriminators (Zade et al., 2024; Pathmasiri et al., 2024; Li et al., 2024). As shown in Figure 9, n-Pentanal-M, ethanol, 2-Propanol-M, 1-Penten-3-ol, (E)-2-Butenal, 3-Methyl butanal-D, 1-Penten-3-one, 2,5-Dimethyl thiophene, alpha-Pinene, 2-methyl-3-ketotetrahydrofuran-D, Propanal-M, 3-Methyl butanal-M, 1-Pyrroline, 2-acetyl, gamma-Butyrolactone-D, 1-propanol-D, 1-Hydroxy-2-propanone-D, 2-Pentanol, methyl 2-furoate, theaspirane, Butanol-M, 1-propanol-M, 2-Propanol-D, 2-Methyl propanal, (Z)-2-Penten-1-ol, 3-Methyl-3-buten-1-ol, 1-Octen-3-one, 2-Butanone, 1-octanal-D, ethyl benzene, (Z)-2-pentenal, Butanol-D. VIP ≥1 (marked in red) may be the main differential indicator metabolites among different drying methods. These metabolites play an important role in distinguishing POR samples with different drying methods and are the main marker metabolites. It should be noted that some metabolites with high relative abundance in the Gallery Plot, such as (E)-2-heptenal and ethyl hexanoate, had VIP <1 in the PLS-DA model. This indicates that relative abundance and discriminatory power are not equivalent. The Gallery Plot reflects group-specific abundance differences, whereas VIP reflects the contribution of each metabolite to the overall discrimination among all five groups. (As shown in Figure 10).

**FIGURE 9:**
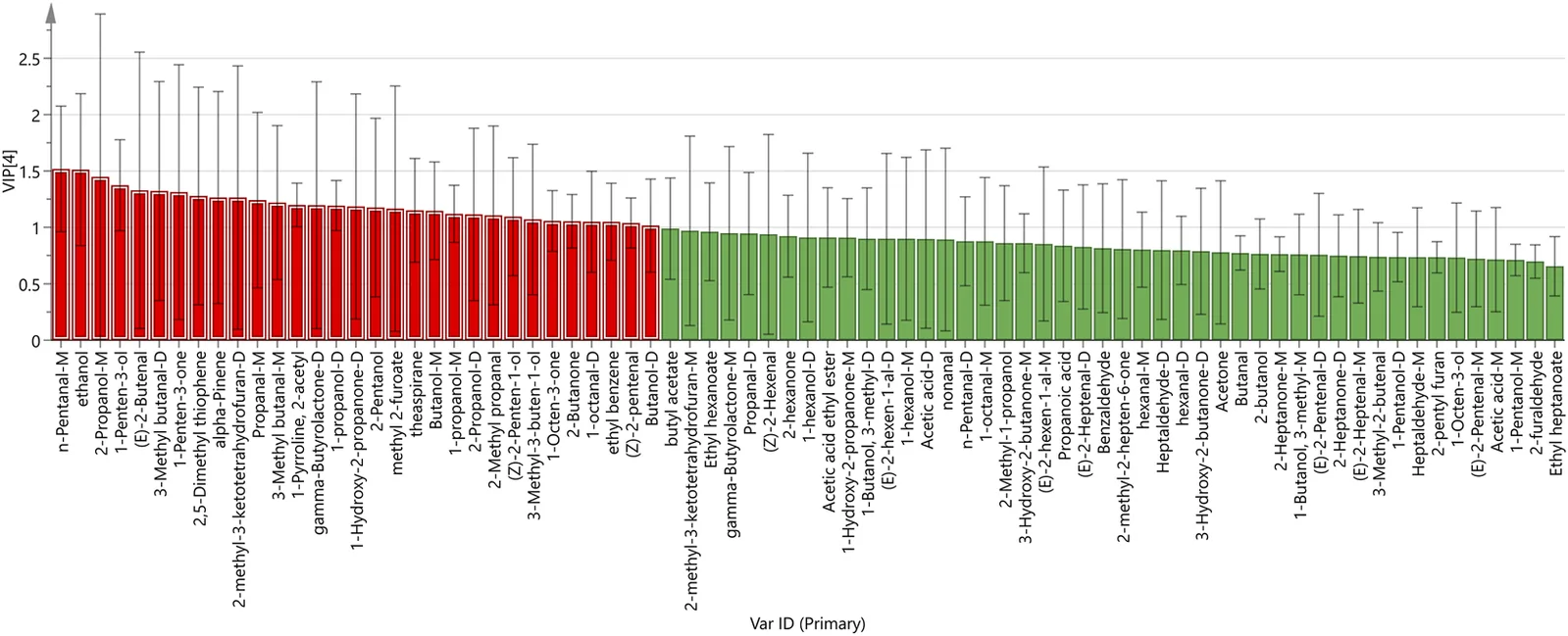
VIP values of the characteristic variables.

**FIGURE 10:**
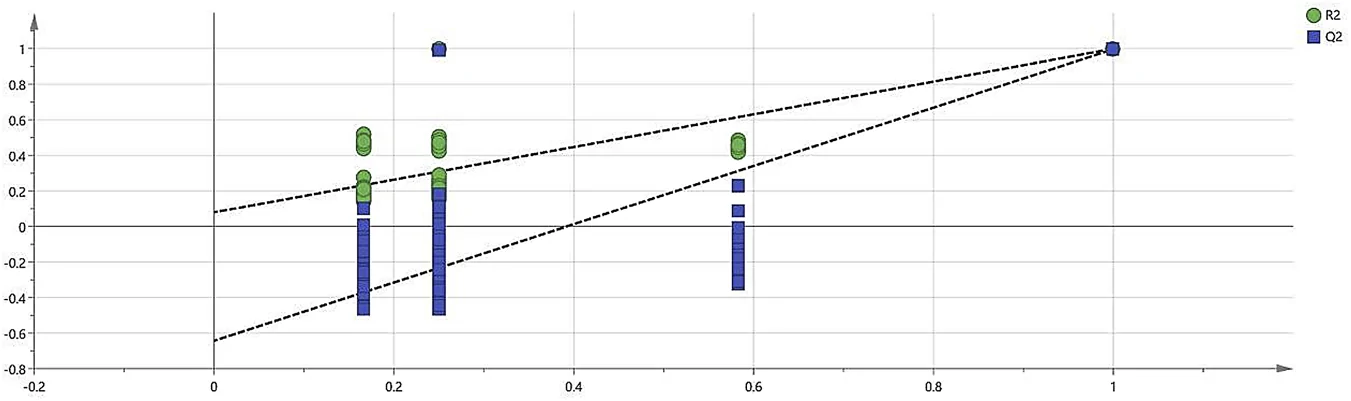
Permutation test results of VOCs in five groups of POR. The original model is shown in the upper right.

### 3.4 Relative odor activity values (ROAV) of VOCs

To assess the impact of VOCs on the overall aroma profile of POR under various drying methods, relative odor activity value (ROAV) analysis was performed. The relative odor activity values (ROAV) of key VOCs (ROAV≥1) in POR samples under different drying methods are presented in Table 2.

**TABLE 2:** VOCs (ROAV ≥1) in POR.

Count	Metabolites	Cas	Formula	Threshold	Flavor description	ROAV				
						YZ-01	YZ-02	YZ-03	YZ-04	YZ-05
1	Nonanal	C124196	C_9_H_18_O	0.001100	Rose, citrus, strong oily	6.96	3.83	2.07	4.45	2.74
2	1-Hexanol-M	C111273	C_6_H_14_O	0.005600	Fresh, fruity, wine, sweet, green	3.34	1.44	0.78	2.02	0.65
	1-Hexanol-D									
3	1-Octanal-M	C124130	C_8_H_16_O	0.000800	Aldehyde, waxy, citrus, orange, fruity, fatty	12.34	4.36	3.80	6.73	3.67
	1-Octanal-D									
4	2-Pentyl furan	C3777693	C_9_H_14_O	0.005800	Bean, fruity, earthy, green, vegetable	1.59	0.78	1.09	0.77	0.84
5	3-Hydroxy-2-butanone-M	C513860	C_4_H_8_O_2_	0.014000	Butter, cream	2.43	9.64	3.32	21.17	3.43
	3-Hydroxy-2-butanone-D									
6	1-Butanol, 3-methyl-M	C123513	C_5_H_12_O	0.004000	Whiskey, banana, fruity	5.18	10.95	4.92	17.89	5.93
	1-Butanol, 3-methyl-D									
7	Hexanal-M	C66251	C_6_H_12_O	0.002400	Fresh, green, fat, fruity	100.00	100.00	100.00	100.00	100.00
	Hexanal-D									
8	Propanal-M	C123386	C_3_H_6_O	0.015100	Pungent, green grassy	3.78	4.10	3.60	5.17	3.24
	Propanal-D									
9	3-Methyl butanal-M	C590863	C_5_H_10_O	0.001100	Chocolate, fat	28.91	37.46	36.05	56.02	31.42
	3-Methyl butanal-D									
10	Acetic acid ethyl ester	C141786	C_4_H_8_O_2_	0.005000	Fresh, fruity, sweet, grassy	2.02	8.57	0.43	15.20	1.06
11	Butanal	C123728	C_4_H_8_O	0.002200	Pungent, fruity, green leaf	4.93	2.04	3.55	2.03	2.26
12	n-Pentanal-M	C110623	C_5_H_10_O	0.012000	Green grassy, faint banana, pungent	4.10	2.49	4.42	3.25	4.46
	n-Pentanal-D									
13	(E)-2-Butenal	C123739	C_4_H_6_O	0.000300	Null	17.36	20.70	15.88	29.82	7.76
14	1-Octen-3-one	C4312996	C_8_H_14_O	0.000016	Strong earthy, mushroom, vegetable, fishy, chicken	25.97	26.39	12.62	62.44	9.78
15	Methyl 2-furoate	C611132	C_6_H_6_O_3_	0.010000	Mushroom, fruity	0.15	0.50	0.22	5.27	0.46
16	1-Pyrroline, 2-acetyl	C85213225	C_6_H_9_NO	0.000053	Popcorn, white bread crust	17.75	35.63	33.63	88.63	18.05
17	Ethyl hexanoate	C123660	C_8_H_16_O_2_	0.002200	Pineapple, fruity, wine	2.06	1.66	1.20	1.36	0.58
18	Heptaldehyde-M	C111717	C_7_H_14_O	0.002800	Fresh, aldehyde, fatty, green herbs, wine, fruity	14.29	9.13	7.18	9.41	8.14
	Heptaldehyde-D									
19	2-Methyl propanal	C78842	C_4_H_8_O	0.000350	Banana, melon, slightly nutty	6.29	23.83	21.35	46.44	16.10
20	2,5-Dimethyl thiophene	C638028	C_6_H_8_S	0.001000	Nutty, sulfury	1.08	2.50	0.91	3.19	1.28

Among the five drying methods, hexanal is a common dominant metabolite with ROAV = 100, indicating that it is one of the most important aroma contributors in POR dried samples, mainly contributing fresh, green, fat and fruity aromas. In addition to hexanal, In the YZ-01 sample (SD), 3-methylbutanal (chocolate, fat), 1-Octen-3-one (strongly earthy, mushroom, vegetable, fishy, chicken), 1-Pyroline, 2-acetyl (popcorn, white bread crust) are the main contributing aroma metabolites; In the YZ-02 sample (HAD), 3-methylbutanal (chocolate, fat) and 1-Pyrroline, 2-acetyl (popcorn, white bread crust) made outstanding contributions. 2-Methylpropanol (banana, melon, light nut) and (E) -2-Butynal (ROAV = 20.70) also have high ROAV; 3-Methylbutanal (chocolate, fat) and 1-Pyrroline, 2-acetyl (popcorn, white bread crust) are the main contributors in the YZ-03 sample (MD); In the YZ-04 sample (VD), 1-Pyrroline, 2-acetyl (popcorn, white bread crust), and 1-Octen-3-one (strongly soil, mushroom, vegetable, fishy, chicken) made outstanding contributions. 3-Methylbutanal (chocolate, fat) and 2-Methylpropanol (banana, melon, light nut) are also higher; 3-Methylbutanal (chocolate, fat) and 1-Pyrroline, 2-acetyl (popcorn, white bread crust) are the main contributors in the YZ-05 sample (VFD).

Overall, YZ-01 sample has a fresh, green, and fruity aroma, with hints of mushrooms, soil, chocolate, and fat; The YZ-02 and YZ-03 samples mainly consist of green, chocolate, fat, popcorn, and fruity flavors; The aroma of the YZ-04 sample is the most rich and complex, with characteristics of green, mushroom, soil, popcorn, chocolate, cream, and fruity aroma; The YZ-05 sample is mainly characterized by green, chocolate, fat, popcorn, and a slight mushroom aroma. Different drying methods have a certain impact on the retention and transformation of key aroma metabolites in POR.

## 4 Discussion

In this study, we have systematically analyzed the regulation of VOCs in POR using five processes, VD, HAD, SD, MD, and FD, using GC-IMS technology. Compared to traditional GC-MS, GC-IMS has outstanding advantages such as no need for complex sample pretreatment, fast analysis speed, and high sensitivity, making it particularly suitable for comparative research on drying processes that require rapid screening of a large number of samples (Ge et al., 2026; Li et al., 2026). The limitation of GC-IMS lies in the fact that its qualitative identification capability is highly dependent on the completeness of the built-in database. For unknown metabolites or isomers not represented in the database, accurate identification by GC-IMS alone is difficult, and confirmation by GC-MS is typically required (Chen et al., 2024). This study identified a total of 56 VOCs, among which aldehydes (32.1%), alcohols (25.0%), and ketones (16.1%) were the dominant metabolites. The drying process also considerably influenced the distribution features of these metabolites. The contents of (E)-2-Pentenal, Ethyl heptanoate, theaspirane, 1-Octen-3-ol, 1-octanal, Ethyl hexanoate, (E)-2-hexen-1-al, Heptaldehyde, (E)-2-Heptenal, nonanal, 2-methyl-2-hepten-6-one, 1-hexanol, and (Z)-2-Hexenal were found to be more abundant in the YZ-01 sample. The content of 2,5-Dimethyl thiophene was found to be more abundant in the YZ-02 sample. The contents of 3-Methyl butanal, n-Pentanal, Propanal, hexanal, 2-hexanone, 1-Penten-3-one, Butanol, 2-Methyl propanal, and (E)-2-Butenal were found to be more abundant in the YZ-03 sample. Acetic acid ethyl ester, Propanoic acid, 2-methyl-3-ketotetrahydrofuran, gamma-Butyrolactone, methyl 2-furoate, and other substances were found to be more abundant in the YZ-04 sample. The contents of 3-Methyl-3-buten-1-ol, (Z)-2-Penten-1-ol, and 2-Pentanol were found to be more abundant in the YZ-05 sample. From the VIP results, it can be seen that n-Pentanal-M, ethanol, 2-Propanol-M, 1-Penten-3-ol, (E)-2-Butenal, 3-Methyl butanal-D, 1-Penten-3-one, 2,5-Dimethyl thiophene, alpha-Pinene, 2-methyl-3-ketotetrahydrofuran-D, Propanal-M, 3-Methyl butanal-M, 1-Pyrroline, 2-acetyl, gamma-Butyrolactone-D, 1-propanol-D, 1-Hydroxy-2-propanone-D, 2-Pentanol, methyl 2-furoate, theaspirane, Butanol-M, 1-propanol-M, 2-Propanol-D, 2-Methyl propanal, (Z)-2-Penten-1-ol, 3-Methyl-3-buten-1-ol, 1-Octen-3-one, 2-Butanone, 1-octanal-D, ethyl benzene, (Z)-2-pentenal, Butanol-D, and other VOCs are the key discriminatory markers for distinguishing POR samples processed by different drying methods.

Our findings are consistent with several directly comparable studies. In ginseng, five drying methods (ND, HAD, VD, MVD, and VFD) were compared using GC-IMS, and 29 VOCs were identified as characteristic markers of different drying methods (Xiang et al., 2024). In Asparagus cochinchinensis roots, HS-GC-IMS revealed that drying method markedly affected the volatile profile (Chen et al., 2022). A multi-platform study on Citrus reticulata ‘Chachi’ peel further showed that vacuum-freeze drying retained the highest VOC content compared with sun-drying and hot-air drying (Wang et al., 2023a). These studies support our observation that drying method is a major determinant of VOC profiles. Within the genus Polygonatum, HS-GC-IMS has been used to discriminate three Polygonati Rhizoma species and their producing areas (Song et al., 2022), and untargeted GC-MS combined with FT-NIR has been applied to compare 14 processing methods of P. kingianum, showing that multiple steaming-roasting samples had higher levels of sugars, nucleotides, and esters but lower levels of amino acids (Xu et al., 2023). A recent HS-GC-IMS study on P. cyrtonema identified 57 VOCs during steaming and screened 18 VIP >1 markers (Shen et al., 2025). In addition, the nine-cycle steam-sun drying of P. cyrtonema has been shown to alter polysaccharides, saponins, and antioxidant activity (Zhu et al., 2022). However, most of these studies focused on species discrimination, processing stages, or non-volatile metabolitess, and few have systematically compared the effects of different drying methods on the VOCs of POR. Our study therefore extends this body of work by providing a direct GC-IMS comparison of five drying methods for POR.

The SD group showed higher relative abundance of several aldehydes and ketones, including (E)-2-heptenal, ethyl heptanoate, and theaspirane. The SD group showed higher relative abundance of (E)-2-heptenal and ethyl hexanoate in the Gallery Plot. (E)-2-Heptenal has been reported to inhibit the activity of Aspergillus flavus (Duan et al., 2023), while ethyl hexanoate has potential anti-obesity effects (Horii et al., 2020). The HAD group showed higher levels of 2,5-dimethylthiophene, while the MD group showed higher levels of 3-methylbutanal, hexanal, and (E)-2-hexenal. Butanal and other short-chain aldehydes were found at higher levels. Among these, (E)-2-butenal has been reported to inhibit the growth of Penicillium expansum and the production of patulin (Taguchi et al., 2013). 2-Butanol was more abundant in the VD group; it has a fruity aroma and is a potential biofuel (Chen et al., 2015). 3-Methyl-3-buten-1-ol was enriched in the VFD group, and research has shown that it may be an important metabolite in nutritional supplements for the elderly (Pandey et al., 2019). After PCA and PLS-DA analyses, it was confirmed that the distance between the VFD group (YZ-05) and the MD group (YZ-03) was relatively small, and these two groups were close to the SD group (YZ-01). This finding suggests that VFD and MD may be promising candidates for further evaluation as alternatives to conventional SD techniques in the large-scale manufacture of POR. The moisture content of the VFD and MD samples was comparable to that of the SD samples. However, the VD group (YZ-04) and the HAD group (YZ-02) were located at a greater distance from the SD group (YZ-01). According to the CA diagram analysis, the SD group, VD group, and HAD group are the last to co-polymerize into a single large cluster. This finding indicates, compared with these two groups, the VOCs of the processed POR samples in the SD group is markedly distinct. Therefore, these two drying methods may not be suitable alternatives to traditional SD. The findings are corroborated by both the PCA and fingerprint spectra, which are consistent with the results of clustering heatmap and PLS-DA analysis. Collectively, these findings indicate that VOCs are not only key determinants of the characteristic aroma and flavor that influence consumer preference and commercial value of POR, but also directly contribute to its diverse pharmacological activities. Given this dual role, the variations in VOCs induced by different drying methods may have significant implications for both the sensory quality and functional properties of the final product. Therefore, elucidating the regulatory effects of drying processes on VOCs is of practical importance for optimizing processing techniques and ensuring the overall quality of POR.

This study has the following limitations: No technical GC-IMS replicates were performed, so instrument precision could not be assessed; in addition, the small sample size (n = 3 per treatment) may inflate the H statistic in the Kruskal–Wallis test and lead to discrete p values, so the univariate statistical results should be interpreted with caution. In addition, Polysaccharides are established pharmacopoeia quality markers for Polygonati Odorati Rhizoma, closely tied to its medicinal efficacy (Lin et al., 2011), the study only focused on VOCs and did not evaluate functional activity, rehydration ability, microstructure, energy consumption, and economic cost, nor did it quantify non-volatile pharmacological markers such as polysaccharides and proteins; The sample source is single, the drying method and batch are mixed, and the multivariate analysis results can only be considered as processing related differences; The VOC characteristics are also affected by extraction techniques, temperature, time, solvent type, sample to solvent ratio, and other factors; The reported antibacterial or anti-obesity activities only target some VOCs, and the functional activities of other VOCs have not been experimentally validated; 80 °C headspace incubation may trigger the Maillard reaction, making it difficult to distinguish whether metabolites originate from drying or incubation; Lack of fresh and undried baseline and intermediate time points, the observed differences cannot be fully attributed to the drying method; No equivalence or non-inferiority criteria have been defined, and microbial safety, sensory quality, energy consumption, cost, flux, and shelf stability have not been evaluated. High R^2^ Y and Q^2^ may overfit due to limited sample size; The aroma description and ROAV are semi quantitative results that have not been validated by sensory panels or GC-O. Therefore, VFD and MD can only be considered as promising candidate methods, rather than confirmed alternatives to SD. Future research should prioritize defining equivalence or non-inferiority criteria, evaluating moisture, microbial safety, non-volatile biomarkers, sensory quality, and energy consumption, and combining them with electronic noses GC-MS,HS-SPME-GC-MS, Sensory evaluation, rehydration and microstructure measurement, as well as *in vitro* and *in vivo* activity determination, establish a “processing method flavor quality functional activity” relationship to develop POR products that combine high consumer acceptance and health benefits.

## 5 Conclusion

In this study, GC-IMS coupled with chemometric techniques to conduct a comprehensive comparative analysis of the VOCs of POR samples subjected to different drying methods. A total of 56 VOCs were identified in five types of POR samples, with aldehydes and alcohols being the most abundant classes. Although POR samples treated with different drying methods share many common VOCs, drying-specific metabolites such as 2,5-dimethyl thiophene (exclusively present in HAD samples) were also observed, and the contents of shared metabolites varied considerably. PCA, CA, and PLS-DA consistently confirmed that the VOCs differed among the five drying treatments. According to the VIP scores, n-pentanal, benzaldehyde, theaspirane, and several other metabolites contributed most to the discrimination among drying methods. Based on the combined results of PCA, CA, and PLS-DA, the VOCs composition of the vacuum freeze-dried and microwave-dried POR samples was comparable to that of traditional sun-dried samples. Therefore, within the VOC dimension, VFD and MD are promising candidates for further evaluation as alternatives to traditional SD.

## Data Availability

The original contributions presented in the study are included in the article/Supplementary Material, further inquiries can be directed to the corresponding author.
