# Supplementary material for: A comparative analysis of the effect of different drying treatments on the volatile organic compounds of polygonati odorati rhizoma
Source: Front Pharmacol. 2026 Oct 15;17:1965242. doi: 10.3389/fphar.2026.1965242 (PMC13716853; doi:10.3389/fphar.2026.1965242)
Supplement: Supplementary file 1 [file Table1.docx]

**TABLE S1** Peak volumes and RSDs of VOCs in POR under five drying methods.

| Count | Metabolite | CAS | YZ-01 | | YZ-02 | | YZ-03 | | YZ-04 | | YZ-05 | |
| --- | --- | --- | --- | --- | --- | --- | --- | --- | --- | --- | --- | --- |
|  |  |  | Average peak volume | RSD | Average peak volume | RSD | Average peak volume | RSD | Average peak volume | RSD | Average peak volume | RSD |
| 1 | Benzaldehyde | C100527 | 271.6199 | 0.02 | 192.4322 | 0.01 | 208.0204 | 0.03 | 190.0015 | 0.05 | 189.9445 | 0.02 |
| 2 | theaspirane | C36431728 | 256.5530 | 0.04 | 222.3956 | 0.05 | 174.7450 | 0.05 | 162.7731 | 0.09 | 191.6131 | 0.07 |
| 3 | Acetic acid-M | C64197 | 4960.4143 | 0.10 | 7366.2442 | 0.06 | 5879.4869 | 0.01 | 9738.3213 | 0.03 | 6071.4813 | 0.10 |
|  | Acetic acid-D | C64197 | 1087.4261 | 0.20 | 2726.9147 | 0.15 | 1608.6883 | 0.12 | 6618.4796 | 0.10 | 1652.9291 | 0.25 |
| 4 | nonanal | C124196 | 466.1213 | 0.04 | 180.6003 | 0.06 | 153.5334 | 0.04 | 143.3552 | 0.03 | 171.8811 | 0.09 |
| 5 | 1-hexanol-M | C111273 | 969.2854 | 0.02 | 301.4870 | 0.07 | 258.0342 | 0.05 | 290.6334 | 0.03 | 174.8561 | 0.02 |
|  | 1-hexanol-D | C111273 | 170.9043 | 0.04 | 42.6566 | 0.07 | 35.8201 | 0.08 | 39.8520 | 0.11 | 33.1036 | 0.10 |
| 6 | (E)-2-Heptenal-M | C18829555 | 1013.1367 | 0.02 | 301.4004 | 0.01 | 560.3885 | 0.01 | 199.3398 | 0.01 | 325.5107 | 0.03 |
|  | (E)-2-Heptenal-D | C18829555 | 120.3913 | 0.04 | 22.1937 | 0.11 | 43.5357 | 0.01 | 19.8704 | 0.17 | 23.3816 | 0.17 |
| 7 | 1-Octen-3-ol | C3391864 | 77.5828 | 0.01 | 44.0068 | 0.05 | 48.3800 | 0.10 | 30.3700 | 0.06 | 48.0119 | 0.07 |
| 8 | Ethyl heptanoate | C106309 | 91.5055 | 0.07 | 52.8836 | 0.01 | 67.6091 | 0.06 | 34.0493 | 0.04 | 60.2638 | 0.17 |
| 9 | 1-octanal-M | C124130 | 553.9311 | 0.05 | 119.4411 | 0.04 | 183.2909 | 0.02 | 119.7470 | 0.03 | 147.2086 | 0.05 |
|  | 1-octanal-D | C124130 | 47.3521 | 0.02 | 29.9279 | 0.04 | 21.4909 | 0.14 | 37.9145 | 0.06 | 20.3355 | 0.11 |
| 10 | 1-Pentanol-M | C71410 | 2239.1617 | 0.01 | 1132.6940 | 0.03 | 1962.5879 | 0.02 | 678.0158 | 0.02 | 1400.0273 | 0.01 |
|  | 1-Pentanol-D | C71410 | 726.1914 | 0.02 | 167.4234 | 0.04 | 484.9697 | 0.05 | 62.2953 | 0.09 | 220.7011 | 0.03 |
| 11 | 2-pentyl furan | C3777693 | 560.7832 | 0.01 | 192.9654 | 0.03 | 423.6232 | 0.06 | 130.9893 | 0.03 | 278.6727 | 0.04 |
| 12 | 1-Hydroxy-2-propanone-M | C116096 | 739.9304 | 0.05 | 1240.2862 | 0.05 | 1134.1952 | 0.02 | 1736.2057 | 0.01 | 705.0368 | 0.09 |
|  | 1-Hydroxy-2-propanone-D | C116096 | 17.2509 | 0.06 | 25.1695 | 0.17 | 45.9427 | 0.09 | 99.9106 | 0.02 | 12.6266 | 0.18 |
| 13 | 3-Hydroxy-2-butanone-D | C513860 | 395.1735 | 0.07 | 2145.7069 | 0.09 | 706.6335 | 0.09 | 4615.6547 | 0.04 | 578.4066 | 0.15 |
|  | 3-Hydroxy-2-butanone-M | C513860 | 1677.0462 | 0.05 | 3636.8405 | 0.02 | 2417.3973 | 0.03 | 4058.8529 | 0.01 | 2161.4212 | 0.07 |
| 14 | (E)-2-hexen-1-al-M | C6728263 | 3708.0630 | 0.00 | 1206.3287 | 0.01 | 1117.8434 | 0.02 | 520.0655 | 0.00 | 1512.6933 | 0.01 |
|  | (E)-2-hexen-1-al-D | C6728263 | 2734.3555 | 0.00 | 246.4504 | 0.03 | 193.6431 | 0.02 | 63.7602 | 0.08 | 307.2222 | 0.04 |
| 15 | 1-Butanol, 3-methyl-M | C123513 | 1093.7167 | 0.01 | 1550.6856 | 0.01 | 1188.6742 | 0.02 | 1723.4873 | 0.00 | 1210.3509 | 0.03 |
|  | 1-Butanol, 3-methyl-D | C123513 | 167.7611 | 0.01 | 325.4441 | 0.01 | 135.2545 | 0.05 | 370.6677 | 0.02 | 144.0618 | 0.02 |
| 16 | Butanol-M | C71363 | 753.7375 | 0.02 | 552.4137 | 0.02 | 888.4314 | 0.02 | 593.7661 | 0.00 | 717.9463 | 0.02 |
|  | Butanol-D | C71363 | 135.1071 | 0.03 | 75.9528 | 0.02 | 154.8198 | 0.04 | 71.6476 | 0.02 | 86.9359 | 0.02 |
| 17 | ethyl benzene | C100414 | 100.0180 | 0.05 | 46.2026 | 0.03 | 53.1458 | 0.03 | 63.7224 | 0.03 | 39.5876 | 0.04 |
| 18 | hexanal-M | C66251 | 5075.2320 | 0.01 | 3916.8463 | 0.01 | 5468.1843 | 0.00 | 3238.4336 | 0.00 | 4971.7833 | 0.01 |
|  | hexanal-D | C66251 | 9540.0510 | 0.01 | 6361.1926 | 0.01 | 10682.3407 | 0.01 | 3784.8386 | 0.01 | 8726.2583 | 0.01 |
| 19 | (Z)-2-pentenal | C1576869 | 61.4154 | 0.02 | 48.3963 | 0.03 | 27.5890 | 0.03 | 65.8768 | 0.02 | 27.5046 | 0.05 |
| 20 | 1-Penten-3-ol | C616251 | 823.3811 | 0.02 | 803.5654 | 0.02 | 838.5634 | 0.02 | 600.9270 | 0.01 | 718.4551 | 0.02 |
| 21 | Propanal-M | C123386 | 928.6196 | 0.03 | 993.6195 | 0.02 | 881.4445 | 0.07 | 944.6589 | 0.01 | 1059.8341 | 0.01 |
|  | Propanal-D | C123386 | 2543.7126 | 0.00 | 1654.8058 | 0.02 | 2775.2199 | 0.01 | 1337.8342 | 0.00 | 1734.7489 | 0.02 |
| 22 | Acetone | C67641 | 5887.6395 | 0.00 | 3426.2576 | 0.01 | 3647.3876 | 0.03 | 2846.5876 | 0.00 | 3212.9411 | 0.00 |
| 23 | ethanol | C64175 | 10816.4063 | 0.00 | 11606.4157 | 0.00 | 10901.2323 | 0.05 | 11164.0547 | 0.00 | 10365.7753 | 0.01 |
| 24 | 2-Butanone | C78933 | 495.1345 | 0.07 | 330.8216 | 0.02 | 219.2732 | 0.04 | 550.5740 | 0.02 | 122.9945 | 0.03 |
| 25 | 2-Propanol-D | C67630 | 529.7563 | 0.01 | 709.7759 | 0.01 | 513.2468 | 0.03 | 674.9749 | 0.01 | 587.4576 | 0.01 |
|  | 2-Propanol-M | C67630 | 65.9938 | 0.02 | 65.3480 | 0.05 | 48.4556 | 0.10 | 61.6576 | 0.01 | 85.0466 | 0.04 |
| 26 | 3-Methyl butanal-M | C590863 | 406.3182 | 0.02 | 349.6411 | 0.00 | 398.7647 | 0.04 | 340.4606 | 0.01 | 469.7303 | 0.02 |
|  | 3-Methyl butanal-D | C590863 | 1530.1368 | 0.01 | 1414.8097 | 0.02 | 2269.4562 | 0.02 | 1462.9550 | 0.01 | 1503.0196 | 0.00 |
| 27 | Acetic acid ethyl ester | C141786 | 614.4275 | 0.02 | 1834.8314 | 0.01 | 143.2678 | 0.02 | 2223.9401 | 0.01 | 301.7603 | 0.02 |
| 28 | Butanal | C123728 | 660.1302 | 0.10 | 192.0041 | 0.04 | 526.0674 | 0.03 | 130.9338 | 0.02 | 283.5266 | 0.02 |
| 29 | n-Pentanal-M | C110623 | 594.4452 | 0.02 | 489.3511 | 0.01 | 796.6089 | 0.02 | 677.3359 | 0.01 | 1020.9915 | 0.04 |
|  | n-Pentanal-D | C110623 | 2399.5294 | 0.02 | 788.7022 | 0.01 | 2774.7695 | 0.01 | 465.3392 | 0.01 | 2030.9843 | 0.01 |
| 30 | (E)-2-Butenal | C123739 | 317.2226 | 0.02 | 265.9417 | 0.03 | 320.6850 | 0.02 | 261.7565 | 0.01 | 132.8483 | 0.02 |
| 31 | 1-propanol-M | C71238 | 445.9820 | 0.02 | 463.0670 | 0.03 | 296.9901 | 0.03 | 466.0717 | 0.01 | 267.5673 | 0.02 |
|  | 1-propanol-D | C71238 | 321.7544 | 0.02 | 336.5369 | 0.02 | 78.6063 | 0.07 | 300.0947 | 0.03 | 58.8181 | 0.08 |
| 32 | 2-Methyl-1-propanol | C78831 | 64.6652 | 0.12 | 169.1979 | 0.09 | 42.5515 | 0.06 | 381.0361 | 0.03 | 84.3801 | 0.12 |
| 33 | 2-Heptanone-M | C110430 | 517.5341 | 0.02 | 239.5272 | 0.03 | 440.6682 | 0.02 | 198.4518 | 0.01 | 322.4520 | 0.03 |
|  | 2-Heptanone-D | C110430 | 166.3332 | 0.03 | 40.7155 | 0.03 | 89.8228 | 0.08 | 25.9901 | 0.06 | 52.4408 | 0.10 |
| 34 | 1-Octen-3-one | C4312996 | 25.3057 | 0.09 | 18.0811 | 0.05 | 13.5901 | 0.03 | 29.2354 | 0.07 | 8.9354 | 0.09 |
| 35 | methyl 2-furoate | C611132 | 93.3340 | 0.13 | 215.4642 | 0.27 | 146.3139 | 0.09 | 1542.1968 | 0.20 | 260.8063 | 0.46 |
| 36 | 2-furaldehyde | C98011 | 231.9049 | 0.02 | 122.9138 | 0.09 | 193.7327 | 0.06 | 71.9357 | 0.12 | 139.8692 | 0.05 |
| 37 | (Z)-2-Penten-1-ol | C1576950 | 366.8025 | 0.01 | 468.3750 | 0.01 | 447.5573 | 0.01 | 403.4432 | 0.01 | 578.0638 | 0.01 |
| 38 | 1-Pyrroline, 2-acetyl | C85213225 | 57.2821 | 0.13 | 80.8719 | 0.04 | 119.9440 | 0.08 | 137.4689 | 0.09 | 54.6159 | 0.05 |
| 39 | 3-Methyl-3-buten-1-ol | C763326 | 174.0229 | 0.01 | 187.0139 | 0.02 | 186.0637 | 0.09 | 196.9306 | 0.03 | 212.3196 | 0.04 |
| 40 | Ethyl hexanoate | C123660 | 276.3723 | 0.05 | 156.6135 | 0.04 | 177.1972 | 0.06 | 87.8254 | 0.11 | 72.6763 | 0.05 |
| 41 | 3-Methyl-2-butenal | C107868 | 210.6725 | 0.01 | 114.0939 | 0.02 | 182.9443 | 0.00 | 76.4971 | 0.04 | 178.8191 | 0.02 |
| 42 | Heptaldehyde-M | C111717 | 1805.5308 | 0.01 | 942.6784 | 0.01 | 1137.6413 | 0.02 | 690.5676 | 0.01 | 1118.8980 | 0.00 |
|  | Heptaldehyde-D | C111717 | 631.4221 | 0.04 | 151.6093 | 0.01 | 215.7457 | 0.02 | 80.2053 | 0.04 | 181.4364 | 0.01 |
| 43 | (E)-2-Pentenal-M | C1576870 | 504.4254 | 0.01 | 212.8240 | 0.04 | 305.8395 | 0.02 | 151.2027 | 0.02 | 252.7545 | 0.02 |
|  | (E)-2-Pentenal-D | C1576870 | 179.8945 | 0.02 | 51.9127 | 0.08 | 77.6776 | 0.02 | 22.1100 | 0.04 | 38.0167 | 0.02 |
| 44 | 2-Pentanol | C6032297 | 246.7963 | 0.01 | 407.7572 | 0.01 | 235.5620 | 0.02 | 378.1100 | 0.02 | 493.9969 | 0.02 |
| 45 | 2-hexanone | C591786 | 276.4479 | 0.05 | 208.7233 | 0.02 | 298.3809 | 0.01 | 74.2057 | 0.04 | 232.6885 | 0.02 |
| 46 | butyl acetate | C123864 | 54.3804 | 0.07 | 81.0363 | 0.07 | 86.7834 | 0.07 | 136.9660 | 0.08 | 46.3108 | 0.04 |
| 47 | 1-Penten-3-one | C1629589 | 92.8630 | 0.03 | 83.2366 | 0.03 | 106.9811 | 0.02 | 69.9553 | 0.01 | 69.0377 | 0.04 |
| 48 | 2-butanol | C78922 | 258.1298 | 0.02 | 696.0444 | 0.02 | 567.6649 | 0.03 | 1057.7115 | 0.01 | 453.0526 | 0.03 |
| 49 | alpha-Pinene | C80568 | 154.2525 | 0.01 | 136.4009 | 0.04 | 72.6882 | 0.17 | 124.6416 | 0.03 | 124.1921 | 0.12 |
| 50 | 2-Methyl propanal | C78842 | 133.9666 | 0.08 | 357.2079 | 0.01 | 502.9634 | 0.03 | 475.6292 | 0.02 | 321.6722 | 0.02 |
| 51 | (Z)-2-Hexenal | R218138 | 216.5900 | 0.06 | 114.7693 | 0.01 | 87.6817 | 0.04 | 97.6303 | 0.03 | 102.4198 | 0.02 |
| 52 | 2,5-Dimethyl thiophene | C638028 | 65.6087 | 0.02 | 107.1308 | 0.04 | 61.3273 | 0.08 | 93.2429 | 0.02 | 73.0214 | 0.04 |
| 53 | gamma-Butyrolactone-M | C96480 | 931.1821 | 0.07 | 1530.9092 | 0.04 | 982.0698 | 0.03 | 3614.3720 | 0.01 | 850.5264 | 0.28 |
|  | gamma-Butyrolactone-D | C96480 | 67.5758 | 0.11 | 84.9481 | 0.03 | 64.1912 | 0.05 | 808.4712 | 0.16 | 70.6885 | 0.12 |
| 54 | Propanoic acid | C79094 | 208.8817 | 0.12 | 272.4901 | 0.05 | 210.9673 | 0.02 | 368.9746 | 0.02 | 168.4766 | 0.07 |
| 55 | 2-methyl-3-ketotetrahydrofuran-M | C3188009 | 37.1517 | 0.04 | 64.5126 | 0.05 | 53.9864 | 0.02 | 153.3053 | 0.02 | 38.1745 | 0.14 |
|  | 2-methyl-3-ketotetrahydrofuran-D | C3188009 | 16.8858 | 0.08 | 15.9741 | 0.10 | 16.8873 | 0.05 | 34.4707 | 0.06 | 16.7947 | 0.22 |
| 56 | 2-methyl-2-hepten-6-one | C110930 | 246.8229 | 0.03 | 62.1819 | 0.04 | 87.7476 | 0.01 | 42.4589 | 0.03 | 62.8359 | 0.08 |

**TABLE S2** statistical analysis of volatile metabolites in POR under five drying methods.

| Compound | p | H | q(FDR) | ε² | 95% CI |
| --- | --- | --- | --- | --- | --- |
| (E)-2-Butenal | 0.0153 | 12.3000 | 0.0181 | 0.879 | 0.735–0.965 |
| (E)-2-Heptenal-D | 0.0221 | 11.4333 | 0.0240 | 0.817 | 0.58–0.958 |
| (E)-2-Heptenal-M | 0.0091 | 13.5000 | 0.0161 | 0.964 | 0.891–0.976 |
| (E)-2-Pentenal-D | 0.0091 | 13.5000 | 0.0161 | 0.964 | 0.891–0.976 |
| (E)-2-Pentenal-M | 0.0091 | 13.5000 | 0.0161 | 0.964 | 0.888–0.976 |
| (E)-2-hexen-1-al-D | 0.0091 | 13.5000 | 0.0161 | 0.964 | 0.891–0.976 |
| (E)-2-hexen-1-al-M | 0.0091 | 13.5000 | 0.0161 | 0.964 | 0.889–0.976 |
| (Z)-2-Hexenal | 0.0091 | 13.5000 | 0.0161 | 0.964 | 0.889–0.976 |
| (Z)-2-Penten-1-ol | 0.0091 | 13.5000 | 0.0161 | 0.964 | 0.891–0.976 |
| (Z)-2-pentenal | 0.0118 | 12.9000 | 0.0161 | 0.921 | 0.751–0.973 |
| 1-Butanol, 3-methyl-D | 0.0091 | 13.5000 | 0.0161 | 0.964 | 0.888–0.976 |
| 1-Butanol, 3-methyl-M | 0.0121 | 12.8333 | 0.0161 | 0.917 | 0.723–0.971 |
| 1-Hydroxy-2-propanone-D | 0.0091 | 13.5000 | 0.0161 | 0.964 | 0.888–0.975 |
| 1-Hydroxy-2-propanone-M | 0.0118 | 12.9000 | 0.0161 | 0.921 | 0.776–0.973 |
| 1-Octen-3-ol | 0.0197 | 11.7000 | 0.0220 | 0.836 | 0.563–0.966 |
| 1-Octen-3-one | 0.0091 | 13.5000 | 0.0161 | 0.964 | 0.888–0.976 |
| 1-Pentanol-D | 0.0091 | 13.5000 | 0.0161 | 0.964 | 0.888–0.976 |
| 1-Pentanol-M | 0.0091 | 13.5000 | 0.0161 | 0.964 | 0.866–0.976 |
| 1-Penten-3-ol | 0.0128 | 12.7000 | 0.0162 | 0.907 | 0.719–0.967 |
| 1-Penten-3-one | 0.0121 | 12.8333 | 0.0161 | 0.917 | 0.726–0.969 |
| 1-Pyrroline, 2-acetyl | 0.0132 | 12.6333 | 0.0164 | 0.902 | 0.772–0.969 |
| 1-hexanol-D | 0.0273 | 10.9333 | 0.0281 | 0.781 | 0.543–0.955 |
| 1-hexanol-M | 0.0118 | 12.9000 | 0.0161 | 0.921 | 0.771–0.969 |
| 1-octanal-D | 0.0121 | 12.8333 | 0.0161 | 0.917 | 0.746–0.971 |
| 1-octanal-M | 0.0121 | 12.8333 | 0.0161 | 0.917 | 0.754–0.969 |
| 1-propanol-D | 0.0102 | 13.2333 | 0.0161 | 0.945 | 0.819–0.974 |
| 1-propanol-M | 0.0146 | 12.4000 | 0.0176 | 0.886 | 0.703–0.971 |
| 2,5-Dimethyl thiophene | 0.0111 | 13.0333 | 0.0161 | 0.931 | 0.804–0.973 |
| 2-Butanone | 0.0091 | 13.5000 | 0.0161 | 0.964 | 0.881–0.976 |
| 2-Heptanone-D | 0.0091 | 13.5000 | 0.0161 | 0.964 | 0.883–0.976 |
| 2-Heptanone-M | 0.0091 | 13.5000 | 0.0161 | 0.964 | 0.89–0.975 |
| 2-Methyl propanal | 0.0091 | 13.5000 | 0.0161 | 0.964 | 0.883–0.975 |
| 2-Methyl-1-propanol | 0.0091 | 13.5000 | 0.0161 | 0.964 | 0.88–0.976 |
| 2-Pentanol | 0.0091 | 13.5000 | 0.0161 | 0.964 | 0.893–0.973 |
| 2-Propanol-D | 0.0111 | 13.0333 | 0.0161 | 0.931 | 0.799–0.975 |
| 2-Propanol-M | 0.0118 | 12.9000 | 0.0161 | 0.921 | 0.759–0.971 |
| 2-butanol | 0.0091 | 13.5000 | 0.0161 | 0.964 | 0.891–0.975 |
| 2-furaldehyde | 0.0091 | 13.5000 | 0.0161 | 0.964 | 0.882–0.976 |
| 2-hexanone | 0.0091 | 13.5000 | 0.0161 | 0.964 | 0.88–0.973 |
| 2-methyl-2-hepten-6-one | 0.0121 | 12.8333 | 0.0161 | 0.917 | 0.739–0.967 |
| 2-methyl-3-ketotetrahydrofuran-D | 0.1257 | 7.2000 | 0.1257 | 0.514 | 0.23–0.934 |
| 2-methyl-3-ketotetrahydrofuran-M | 0.0118 | 12.9000 | 0.0161 | 0.921 | 0.785–0.971 |
| 2-pentyl furan | 0.0091 | 13.5000 | 0.0161 | 0.964 | 0.88–0.975 |
| 3-Hydroxy-2-butanone-D | 0.0102 | 13.2333 | 0.0161 | 0.945 | 0.816–0.974 |
| 3-Hydroxy-2-butanone-M | 0.0091 | 13.5000 | 0.0161 | 0.964 | 0.889–0.974 |
| 3-Methyl butanal-D | 0.0091 | 13.5000 | 0.0161 | 0.964 | 0.884–0.976 |
| 3-Methyl butanal-M | 0.0118 | 12.9000 | 0.0161 | 0.921 | 0.75–0.969 |
| 3-Methyl-2-butenal | 0.0091 | 13.5000 | 0.0161 | 0.964 | 0.889–0.975 |
| 3-Methyl-3-buten-1-ol | 0.0285 | 10.8333 | 0.0289 | 0.774 | 0.517–0.969 |
| alpha-Pinene | 0.0197 | 11.7000 | 0.0220 | 0.836 | 0.599–0.976 |
| gamma -Butyrolactone-D | 0.0273 | 10.9333 | 0.0281 | 0.781 | 0.53–0.958 |
| gamma -Butyrolactone-M | 0.0241 | 11.2333 | 0.0257 | 0.802 | 0.547–0.967 |
| Acetic acid ethyl ester | 0.0091 | 13.5000 | 0.0161 | 0.964 | 0.893–0.975 |
| Acetic acid-D | 0.0118 | 12.9000 | 0.0161 | 0.921 | 0.783–0.972 |
| Acetic acid-M | 0.0118 | 12.9000 | 0.0161 | 0.921 | 0.762–0.973 |
| Acetone | 0.0091 | 13.5000 | 0.0161 | 0.964 | 0.886–0.974 |
| Benzaldehyde | 0.0262 | 11.0333 | 0.0276 | 0.788 | 0.515–0.964 |
| Butanal | 0.0091 | 13.5000 | 0.0161 | 0.964 | 0.892–0.973 |
| Butanol-D | 0.0091 | 13.5000 | 0.0161 | 0.964 | 0.883–0.976 |
| Butanol-M | 0.0091 | 13.5000 | 0.0161 | 0.964 | 0.888–0.976 |
| Ethyl heptanoate | 0.0157 | 12.2333 | 0.0183 | 0.874 | 0.679–0.969 |
| Ethyl hexanoate | 0.0091 | 13.5000 | 0.0161 | 0.964 | 0.876–0.975 |
| Heptaldehyde-D | 0.0091 | 13.5000 | 0.0161 | 0.964 | 0.868–0.975 |
| Heptaldehyde-M | 0.0111 | 13.0333 | 0.0161 | 0.931 | 0.796–0.973 |
| Propanal-D | 0.0091 | 13.5000 | 0.0161 | 0.964 | 0.885–0.974 |
| Propanal-M | 0.0203 | 11.6333 | 0.0223 | 0.831 | 0.529–0.968 |
| Propanoic acid | 0.0144 | 12.4333 | 0.0176 | 0.888 | 0.698–0.973 |
| butyl acetate | 0.0118 | 12.9000 | 0.0161 | 0.921 | 0.785–0.972 |
| ethanol | 0.0197 | 11.7000 | 0.0220 | 0.836 | 0.607–0.971 |
| ethyl benzene | 0.0091 | 13.5000 | 0.0161 | 0.964 | 0.889–0.975 |
| hexanal-D | 0.0091 | 13.5000 | 0.0161 | 0.964 | 0.886–0.976 |
| hexanal-M | 0.0091 | 13.5000 | 0.0161 | 0.964 | 0.869–0.975 |
| methyl 2-furoate | 0.0118 | 12.9000 | 0.0161 | 0.921 | 0.751–0.971 |
| n-Pentanal-D | 0.0091 | 13.5000 | 0.0161 | 0.964 | 0.863–0.975 |
| n-Pentanal-M | 0.0091 | 13.5000 | 0.0161 | 0.964 | 0.896–0.976 |
| nonanal | 0.0125 | 12.7667 | 0.0162 | 0.912 | 0.744–0.971 |
| theaspirane | 0.0128 | 12.7000 | 0.0162 | 0.907 | 0.748–0.971 |

Notes: H, Kruskal-Wallis test statistic; p, raw p value; q (FDR), Benjamini-Hochberg adjusted p value; ε², epsilon-squared effect size; 95% CI, bootstrap confidence interval for ε².
